# Biomimetic total synthesis of ten *Securinega* alkaloids

**DOI:** 10.1039/d5qo01704a

**Published:** 2026-01-13

**Authors:** Lachlan J. N. Waddell, Zhouqian Jiang, Gideon Grogan, Benjamin R. Lichman, William P. Unsworth

**Affiliations:** a University of York, Department of Chemistry Heslington York YO10 5DD UK william.unsworth@york.ac.uk; b University of York, Centre for Novel Agricultural Products, Department of Biology Heslington York YO10 5DD UK benjamin.lichman@york.ac.uk

## Abstract

The *Securinega* alkaloids are a diverse family of polycyclic alkaloids with broad biological importance. In this study, a formal [4 + 2] cycloaddition strategy has been used to complete the biomimetic total synthesis of six *Securinega* alkaloids, notably using biosynthetically plausible scaffold-forming steps and aqueous reaction conditions. A further four alkaloids were also generated *via* subsequent rearrangement reactions. Altogether, the total syntheses of ten *Securinega* alkaloids are described, in one or two linear steps from proposed biosynthetic precursor menisdaurilide. These results support the hypothesis that the same or similar pathways may operate *in planta*.

## Abstract 2

The biomimetic synthesis of ten *Securinega* alkaloids is reported, using biosynthetically relevant starting materials and conditions.

## Graphical abstract

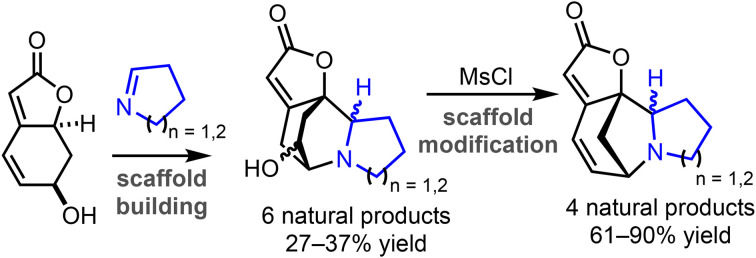

## Introduction

**Fig. 1:**
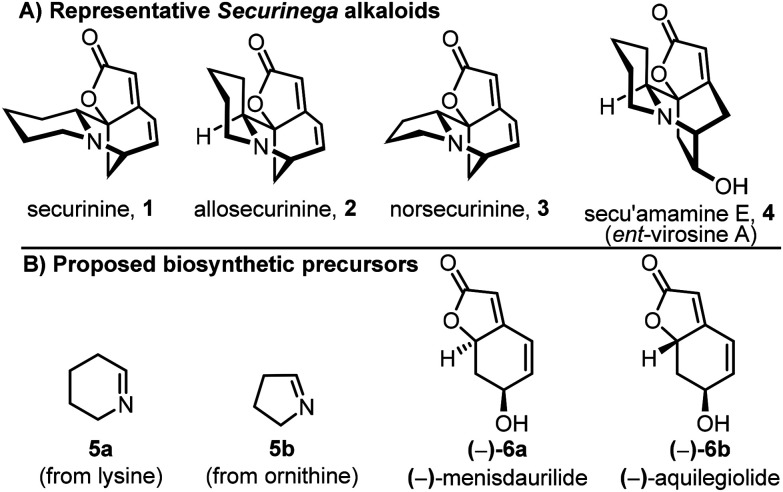
*Securinega* alkaloids and proposed precursors.

*Securinega* alkaloids are a family of structurally diverse polycyclic alkaloids, with over 100 examples reported, isolated from plants from the Phyllanthaceae family, primarily from the genera *Flueggea* and *Phyllanthus*.^1^ Several of these plants have a long history of use in traditional medicine in Africa and Asia,^2^ with numerous pharmacologically relevant *Securinega* alkaloids known. This includes compounds with antimalarial, antitumor and antimicrobial activity.^3^ Securinine (1) – first isolated^1*c*^ from *Flueggea suffruticosa* (formerly *Securinega suffruticosa*) – is the most abundant and well-studied alkaloid in this class, with other prominent members including diastereoisomers (*e.g.* allosecurinine, 2), homologues (*e.g.* norsecurinine, 3) and hydroxylated variants (*e.g.* secu'amamine E (4), also known as *ent*-virosine A, Fig. 1A). The diversity, structural complexity and biological activity of *Securinega* alkaloids has prompted extensive efforts from synthetic chemists to develop methods to prepare them, resulting in the publication of many excellent total syntheses.^4,5^

The biosynthesis of *Securinega* alkaloids has also been investigated.^6,7^ It is well accepted that cyclic imines (Fig. 1B) are likely biosynthetic precursors, with Δ^1^-piperideine 5a being incorporated into piperidine-containing scaffolds (*e.g.* securinine, 1) and 1-pyrroline 5b incorporated into pyrrolidine derivatives (*e.g.* norsecurinine, 3). In seminal work by Parry,^6*d*^ a biosynthesis was proposed in which imine 5a is elaborated *via* an electrophilic aromatic substitution reaction with tyrosine. However, the later discovery of bicyclic butenolide natural products (−)-menisdaurilide (−)-6a and (−)-aquilegiolide (−)-6b in *Securinega* and *Phyllanthus* genus plants^8^ led to Parry's proposal being reconsidered.

**Scheme 1:**
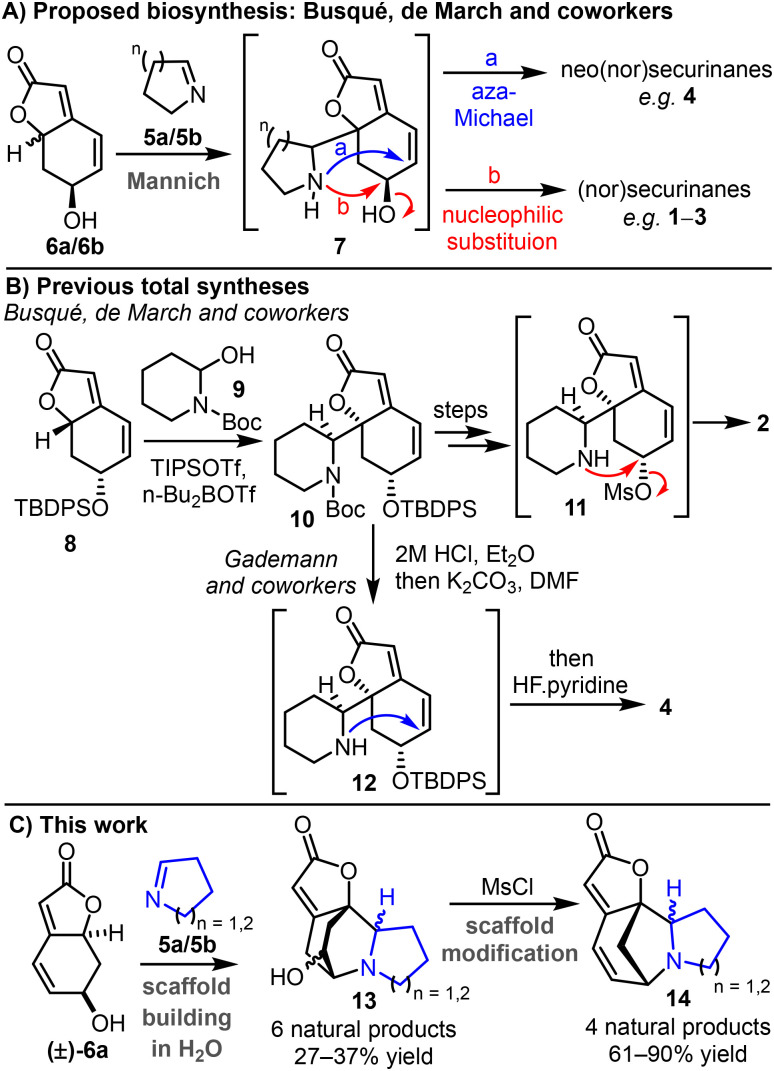
(A) Proposed biosynthesis; (B) previous total syntheses; (C) this work: biomimetic total synthesis of ten *Securinega* alkaloids.

Recognising 6a and 6b as alternative biosynthetic precursors, Busqué, de March and co-workers proposed a revised biosynthesis able to account for the formation of several *Securinega* alkaloid scaffolds (Scheme 1A).^7^ It was proposed that *Securinega* alkaloids may originate from the coupling of the previously proposed imines (5a/5b) with butenolides 6a and 6b*via* a Mannich-type reaction, a typical step in alkaloid formation.^9^ The resulting coupled product (7) could then cyclise by one of two pathways, either undergoing an aza-Michael reaction to produce neo(nor)securinanes (*e.g.*4), or nucleophilic substitution to form (nor)securinanes (*e.g.*1–3).

Support for this biosynthetic proposal is found in subsequent total syntheses (Scheme 1B). For example, Busqué, de March and co-workers showed that protected (+)-menisdaurilide derivative 8 reacts with *N*-acyl iminium ion precursor 9 to form coupled product 10, following sequential activation steps using triisopropylsilyl trifluoromethanesulfonate and dibutylboron trifluoromethanesulfonate.^7^ Protecting group cleavage steps and alcohol activation (mesylation) steps followed, to form intermediate 11, which underwent nucleophilic substitution to form allosecurinine 2. Gademann and coworkers later showed that intermediate 10 from Busqué, de March and co-workers’ route can be converted into secu'amamine E/*ent*-virosine 4 (a neosecurinane) following sequential Boc-cleavage, aza-Michael reaction and removal of the TBDPS protecting group.^10,11^ The bioinspired strategy of Peixoto and coworkers’ is also noteworthy; in this work, menisdaurilide derivatives were trapped with linear azido aldehydes rather than cyclic imines, and subsequent conversion into cyclic amines/imines enabled the total synthesis of twelve *Securinega* alkaloids.^12^

These previous total syntheses provide good support for the proposed pathways summarised in Scheme 1A. However, notably all make use of protecting groups and activating reagents that are not present naturally, and the reactions were performed in organic solvents.^10–12^ In this study, we set out to test whether *Securinega* alkaloids could be generated under more biosynthetically relevant conditions (Scheme 1C). The successful realisation of this idea is reported herein, with six *Securinega* alkaloids synthesised from menisdaurilide (±)-6a and cyclic imines 5a and 5b, with the key scaffold forming steps able to be performed in aqueous conditions, without using protecting groups or activating reagents. Furthermore, another four alkaloids were generated following subsequent rearrangement steps, which also have biosynthetic justification. To the best of our knowledge, the total syntheses described herein represents the shortest general strategy to synthesize *Securinega* alkaloids reported to date. The results also have important biosynthetic implications, corroborating the earlier proposal from Busqué, de March and co-workers,^7^ and supporting the idea that the key scaffold assembly steps of some alkaloids in this family may operate without an enzyme.

## Results and discussion

As the stereochemistry-generating steps in our planned syntheses rely on diastereocontrol, we chose to test our synthetic hypothesis using racemic (±)-menisdaurilide ((±)-6a), which we prepared on gram-scale from 4-hydroxyphenylacetic acid using the efficient method reported by Peixoto and co-workers (see SI for full details).^12,13^ Δ^1^-Piperideine 5a was prepared in a single step from piperidine using the method described by O'Brien and coworkers.^14,15^ With proposed biosynthetic building blocks (±)-menisdaurilide ((±)-6a) and Δ^1^-piperideine 5a in hand, we started by simply combining them in water and stirring for 18 hours at 20 °C (Table 1, entry 1). This simple aqueous reaction directly generated a mixture of three diastereomeric neosecurinane alkaloids: (±)-securinol A ((±)-15), (±)-virosine A ((±)-4) and (±)-virosine B ((±)-16). The three alkaloids were formed in 13% NMR yield (determined by quantitative ^1^H NMR spectroscopy using 1,3,5-trimethoxy benzene as an internal standard), with a significant quantity (32%) of unreacted menisdaurilide (±)-6a also present in this mixture. While this overall yield is modest in preparative terms, the formation of three alkaloids in this way, in water without any additional reagents, is highly notable.

**Table 1:** Optimization of the stepwise formal [4 + 2] cycloaddition reaction^a^

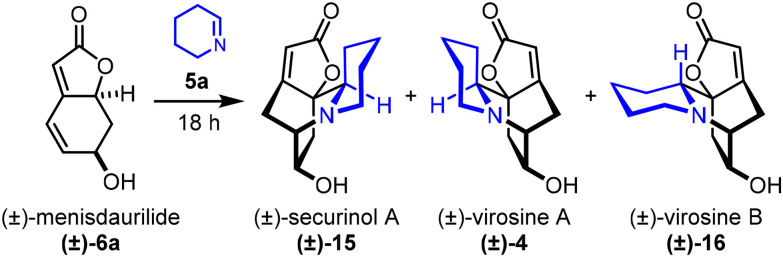				
Entry	Solvent	Additive (equiv.)	Temp. (°C)	Yield (%) (15 : 4 : 16)
1	H_2_O	—	20	13 (6 : 6 : 1)
2	MeOH	—	20	9 (5 : 3 : 1)
3	H_2_O	—	50	19 (6 : 11 : 2)
4	H_2_O	Et_3_N (1.0)	20	19 (7 : 11 : 1)
5	H_2_O	Et_3_N (1.0)	50	17 (6 : 10 : 1)
6	H_2_O	TFA (1.0)	20	0
7	H_2_O	AgOTf (0.2)	20	10 (5 : 4 : 1)
8^b^	H_2_O	Et_3_N (1.0)	20	25 (9 : 15 : 1)
9^b^	H_2_O/THF (3 : 1)	Et_3_N (1.0)	20	33 (14 : 17 : 2)
10^b^^,^^c^	H_2_O/THF (3 : 1)	Et_3_N (1.0)	20	27 (9 : 16 : 2)^c^

a Reaction conditions: (±)-6a (1.0 equiv.) and 5a (1.5 equiv.) were stirred in the specified solvent (0.164 M) for 18 h unless stated otherwise. Yields were determined by quantitative ^1^H NMR spectroscopy, using 1,3,5-trimethoxy benzene as an internal standard.

b 4.5 equiv. 5a.

c Isolated yields after column chromatography.

Optimisation was then performed in an attempt to improve the overall yield of the alkaloids formed (see SI for full details and Table 1 for selected results). In all cases, the % yield for experiments in this series (Table 1 and SI Table S1) were determined by quantitative ^1^H NMR spectroscopy using 1,3,5-trimethoxy benzene as an internal standard. No improvement was observed when methanol was used as the solvent (Table 1, entry 2), while aprotic organic solvents resulted only in the recovery of starting materials (see SI Table S1). In an attempt to drive the reaction to completion, the procedure was conducted at 50 °C, which improved the combined yield of (±)-15, (±)-4 and (±)-16 to 19% (Table 1, entry 3). The addition of one equivalent of triethylamine at 20 °C gave a similar result (19%, Table 1, entry 4), while the combination of these conditions resulted in a slightly reduced yield of 17% (Table 1, entry 5).

Other mild organic and inorganic bases, such as diisopropylethylamine and potassium carbonate, gave similar results, while stronger bases, such as 1,8-diazabicyclo[5.4.0]undec-7-ene and sodium hydroxide, resulted in very low product formation and complete consumption of menisdaurilide (SI Table S1). When organic acids such as trifluoroacetic acid were used, only starting materials were detected (Table 1, entry 6), while Lewis acidic silver triflate mediated a combined yield of 9% (Table 1, entry 7). The best outcome was observed using 4.5 equivalents of 5a (Table 1, entry 8), and tetrahydrofuran as a co-solvent to give a total yield of 33% (entry 9). Using these optimised conditions in a preparative reaction, all three alkaloids were separable by column chromatography (entry 10) to give (±)-15, (±)-4 and (±)-16 in 9%, 16% and 2% isolated yields, respectively.

**Scheme 2:**
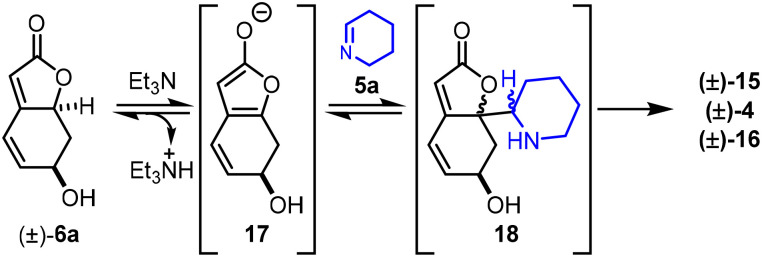
Outline mechanism for the formal [4 + 2] cycloaddition to form (±)-15, (±)-4 and (±)-16.

Alkaloids (±)-15, (±)-4 and (±)-16 are all formal [4 + 2] cycloaddition addition products, presumably formed in a stepwise manner. The improved reactivity observed in the presence of triethylamine is likely due to the base facilitating the formation of the reactive enol/enolate furan tautomer 17. Notably, stronger bases result in decomposition – possibly by polymerisation and/or lactone ring-opening. The reaction is thought to proceed *via* a vinylogous Mannich-type reaction to form intermediate 18, followed by an intramolecular aza-Michael reaction to form (±)-15, (±)-4 or (±)-16 (Scheme 2).

We successfully demonstrated the coupling of 5a and 6a under several different reaction conditions, including a fully aqueous and additive-free reaction. It is therefore plausible that this mechanism (or similar) may also operate *in planta*, without the need for an enzyme to mediate the alkaloid scaffold assembly. Notably, whilst this represents a specific advance for *Securinega* alkaloid (bio)synthesis, it also supports a wider assessment of plant alkaloid biosynthesis in which the key scaffold-forming step for many classes of alkaloids is a Mannich reaction that can proceed without enzyme catalysis.^9^

**Scheme 3:**
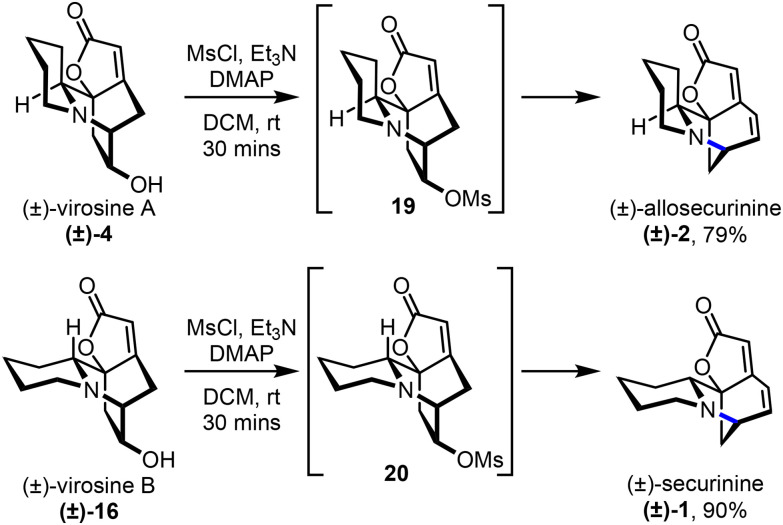
Synthesis of (±)-allosecurinine and (±)-securinine.

While we were conducting this research, Kim and co-workers reported the observation of signals consistent with 15, 4 and 16 by LCMS analysis after incubating 6a with 5a in aqueous buffer in the absence of enzyme.^16^ The same group also discovered a sulfotransferase (*Fs*NSST1/2) able to effect a 1,2-amine shift to form allosecurinine 2 and securinine 1 from alkaloids 4 and 16, which represents a biological equivalent to a rearrangement step already demonstrated by Gademann and co-workers in their total synthesis of 2.^10^ Thus, to expand the number of alkaloids accessible in this study, we treated (±)-4 and (±)-16 with methanesuflonyl chloride in the presence of triethylamine, using Gademann's method, to successfully form (±)-allosecurinine ((±)-2) and (±)-securinine ((±)-1) in 79% and 90% yields, respectively (Scheme 3).

**Scheme 4:**
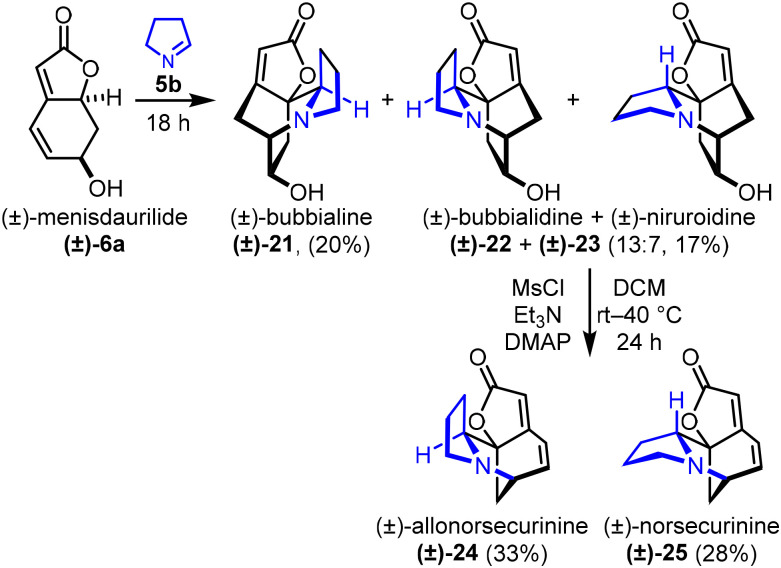
Synthesis of (±)-allonorsecurinine and (±)-norsecurinine.

Next, attention turned to the related neonorsecurinine alkaloids, which feature a 5-membered pyrrolidine ring, thought to be derived biosynthetically from 1-pyrroline (5b). Pleasingly, the reaction between (±)-menidurilide ((±)-6a) and 1-pyrroline (5b) proceeded in similar fashion to the analogous reaction with Δ^1^-piperideine 5a, with (±)-bubbialine ((±)-21) obtained in 20% isolated yield, and (±)-bubbialidine ((±)-22) and (±)-niruroidine ((±)-23) produced in a combined 17% yield as a inseparable mixture in 13 : 7 ratio (Scheme 4). The diastereomeric mixture of (±)-22 and (±)-23 was then converted into (±)-allonorsecurinine ((±)-24) and (±)-norsecurinine ((±)-25) using the same approach described in Scheme 3. These diastereomers were separable by column chromatography with (±)-24 and (±)-25 isolated in 33% and 28% yields, respectively. In the case of (±)-allonorsecurinine (±)-24, the yield was lower than anticipated due to incomplete conversion of the intermediate mesylate, despite using an elevated temperature (40 °C) and extended reaction time (24 h).

**Scheme 5:**
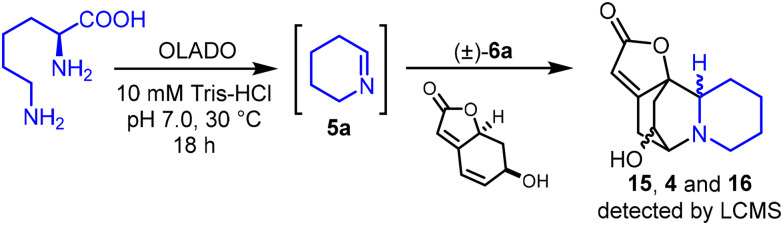
Semi-biocatalytic synthesis of (±)-15, (±)-4 and (±)-16 from l-lysine and menisdaurilide enabled by OLADO.

We have recently discovered and characterized a new class of PLP-dependent enzymes termed OLADOs (ornithine/lysine/arginine decarboxylase-oxidases), which are able to convert lysine to Δ^1^-piperideine (5a), and ornithine to pyrroline (5b).^17^ Thus, we were intrigued by the possibility of the semi-biocatalytic synthesis of 15, 4 and 16, using OLADO to generate 5a from l-lysine in the presence of menisdaurilide (±)-6a. Thus, OLADO was incubated with l-lysine and menisdaurilide in Tris-HCl buffer at pH 7.0 for 18 hours and pleasingly, diastereomeric alkaloids 15, 4 and 16 were each observed by LCMS.^18^ In line with our synthetic studies, 15 and 4 were the most prominent alkaloid products detected (Scheme 5, and SI section S5 for full details).

The outcome of the semi-biocatalytic experiment in Scheme 5 provides further evidence that the general synthetic pathway used in this manuscript is viable in plants. However, it is notable that in plants, virosine B (16) is typically found in higher quantities than 15 and 4 (and other neosecurinanes), and securinine 1 – which we propose derives from 16 – is the most abundant of all the S*ecurinega* alkaloids.^19,20^ This contrasts to our synthetic results (Table 1) and semi-biocatalytic result (Scheme 5) where 16 was the minor diastereomer obtained, being formed in lower quantities than 15 and 4. Of course, many factors influence the abundance and distribution of metabolites in different plants/tissues. Nonetheless, considering this difference, whilst our results provide strong evidence that non-enzymatic alkaloid formation from cyclic imines and 6a*in planta* is viable, they do not rule out the participation of enzymes in alkaloid scaffold assembly operating in parallel.

## Conclusions

In summary, we have synthesized six *Securinega* alkaloids from 4-hydroxyphenylacetic acid and cyclic imines 5a and 5b (*via* menisdaurilide ((±)-6a)), using a stepwise, formal [4 + 2] cycloaddition reaction as a key step. We suggest that the scaffold-forming steps from menisdaurilide ((±)-6a) to the products are not only biomimetic,^21^ but also biosynthetically plausible, both in terms of the reagents used and the aqueous reaction conditions. Another four alkaloids were also generated, by performing subsequent rearrangement steps. While the yields of the individual alkaloids generated *via* the menisdaurilide/imine coupling reactions are modest, the brevity of the route is a major strength, enabling rapid access to analytically pure *Securinega* alkaloids; indeed, in two cases ((±)-15 and (±)-21) our route represents the highest overall yield to these natural products reported to date. In addition to its synthetic utility, the direct formation of six *Securinega* alkaloids from menisdaurilide (±)-6a in water or aqueous buffer, using biologically relevant conditions and without the need for reactive/toxic reagents, has important biosynthetic implications. Most notably, the results corroborate Busqué, de March and co-workers’ previous biosynthetic proposal,^7^ and support a biosynthetic pathway in which a key scaffold-forming step is a Mannich-type reaction, able to proceed without an enzyme.^9^

## Author contributions

G. G., B. J. L. and W. P. U. designed and supervised the study. L. J. N. W. and J. Z. performed the experiments and interpreted the results. L. J. N. W., B. R. L. and W. P. U. prepared the manuscript, supported by all authors.

## Conflicts of interest

There are no conflicts to declare.

## Supplementary Material

QO-013-D5QO01704A-s001

## Data Availability

The data that support the findings of this study are available in the published article and its supplementary information (SI). Supplementary information: detailed experimental procedures and characterization data for new compounds. See DOI: https://doi.org/10.1039/d5qo01704a.
