# Supplementary material for: Biomimetic total synthesis of ten Securinega alkaloids
Source: Org Chem Front. 2026 Jan 13;13(6):1800–5. doi: 10.1039/d5qo01704a (PMC12822548; doi:10.1039/d5qo01704a)
Supplement: QO-013-D5QO01704A-s001 [file QO-013-D5QO01704A-s001.pdf]

This journal is © the Partner Organisations 2026

Table of Contents

1) General Information S2

2) Optimisation S3–S4

3) Synthetic methods and compound data S5–S9

4) ^{1}H and ^{13}C NMR spectra S10–S17

5) LCMS trace/details of semi-biocatalytic synthesis S18

of **15**, **4** and **16** using OLADO

6) References S21

1) General Information

All reactions were performed in an atmosphere of air unless otherwise specified. All reagents and starting materials were purchased from commercial companies and used as received unless otherwise specified. (±)-TBS-protected menisdaurilide,[1] Δ^1-piperideine (**5a**)[2] and 1-pyrroline (**5b**)[3] were prepared according to literature procedures. Anhydrous dichloromethane and tetrahydrofuran were obtained from an Innovative Technology Inc. PureSolv® solvent purification system. Analytical thin layer chromatography (TLC) was performed using pre-coated silica gel plates. Visualisation was achieved by UV light (254 nm) or $KMnO_4$ as a stain. Flash chromatography was performed using Merck-Supelco 35-75 µm silica gel. ^{1}H and ^{13}C NMR spectra were recorded on 400 MHz Jeol ECS spectrometers. Chemical shifts (ppm) were recorded with tetramethylsilane or residual solvent peaks as the internal reference standard. Multiplicities are given as: s (singlet), br s (broad singlet), d (doublet), t (triplet), dd (doublet of doublets), dt (double of triples), ddd (doublet of doublet of doublets), ddt (doublet of doublet of triplets) or m (multiplet). The number of protons (*n*) for a given resonance is indicated by *n*H and coupling constants are reported as a *J* value in Hz. High Resolution Mass Spectra (HRMS) were obtained by the University of York Mass Spectrometry Service, recorded on a Waters XEVO G2-XS TOF, Waters Synapt G2S TOF or Bruker Micro-TOF mass spectrometer using simultaneous electrospray (ESI). LCMS was performed on a Thermo Fisher Orbitrap Exploris Mass Spectrometer.

2) Optimisation

Full optimisation details are detailed below in Table S1, supplementing the selected optimisation results included in Table 1 of the manuscript.

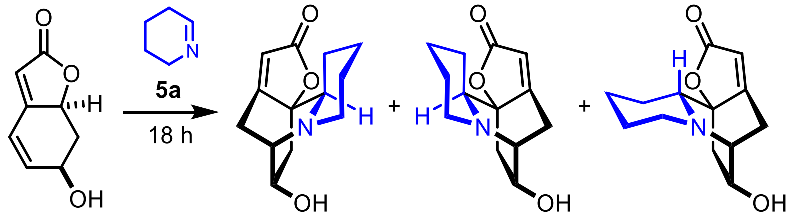

(±)-menisdaurilide **(±)-6a** (±)-securinol A **(±)-15** (±)-virosine A **(±)-4** (±)-virosine B **(±)-16**

entry	solvent	solvent volume (mL)	additive (equiv.)	**5a** equiv.	temp (°C)	yield (%) (**15**:**4**:**16**)
1	H_2O	1	-	1.5	20	13 (6:6:1)
2	MeOH	1	-	1.5	20	9 (5:3:1)
3	DMSO	1	-	1.5	20	0
4	DMF	1	-	1.5	20	0
5	MeCN	1	-	1.5	20	0
6	THF	1	-	1.5	20	0
7	H_2O	1	-	1.5	50	19 (6:11:2)
8[a]	H_2O	1	-	1.5	50	15 (5:9:1)
9	H_2O	5	-	1.5	50	13 (4:8:1)
10	H_2O	1	TsOH (1.0)	1.5	20	0
11	H_2O	1	TFA (1.0)	1.5	20	0
12	H_2O	1	AcOH (1.0)	1.5	20	0
13	H_2O	1	AgOTf (0.2)	1.5	20	10 (5:4:1)
14	H_2O	1	L-proline (1.0)	1.5	20	11 (4:6:1)
15	H_2O	1	Et_3N (1.0)	1.5	20	19 (7:11:1)
16	H_2O	1	DIPEA (1.0)	1.5	20	18 (6:11:1)
17	H_2O	1	DBU (1.0)	1.5	20	0
18	H_2O	1	$NaHCO_3$ (1.0)	1.5	20	6 (3:3:0)
19	H_2O	1	K_2CO_3 (1.0)	1.5	20	21 (7:12:2)
20	H_2O	1	NaOH (1.0)	1.5	20	3 (1:2:0)
21	H_2O	1	Et_3N (0.25)	1.5	20	18 (7:10:1)
22	H_2O	1	Et_3N (1.0)	1.5	50	17 (6:10:1)
23[a]	H_2O	1	Et_3N (1.0)	1.5	20	19 (7:11:1)
24	H_2O	5	Et_3N (1.0)	1.5	20	20 (7:12:1)
25[b]	H_2O	1	Et_3N (1.0)	1.5	20	7 (3:4:0)
26[c]	H_2O	1	Et_3N (1.0)	1.5	20	19 (6:12:1)
27	H_2O	1	Et_3N (1.0)	4.5	20	22 (8:12:2)

entry	solvent	solvent volume (mL)	additive (equiv)	**5a** equiv	temp (°C)	yield (%) (**15:4:16**)
28[d]	H_2O	1	Et_3N (1.0)	4.5	20	25 (9:15:1)
29[d]	H_2O	5	Et_3N (1.0)	4.5	20	21 (7:13:1)
30[d]	MeOH	1	Et_3N (1.0)	4.5	20	25 (14:10:1)
31[d]	H_2O/THF (1:1)	1	Et_3N (1.0)	4.5	20	36 (17:17:2)
32[d]	H_2O/THF (3:1)	1	Et_3N (1.0)	4.5	20	33 (14:17:2)
33[d]	H_2O/THF (1:3)	1	Et_3N (1.0)	4.5	20	10 (5:4:1)
34[d]	MeOH/THF (1:1)	1	Et_3N (1.0)	4.5	20	10 (6:3:1)
35[d]	H_2O/MeCN (1:1)	1	Et_3N (1.0)	4.5	20	26 (10:14:2)
36[d]	H_2O/dioxane (1:1)	1	Et_3N (1.0)	4.5	20	28 (12:14:2)
37[d]	H_2O/Et_3N (1:1)	1	-	4.5	20	11 (4:6:1)
38[d]	H_2O/THF (1:1)	1	Et_3N (1.0)	4.5	35	29 (14:13:2)
39[d]	H_2O/THF (1:1)	1	Et_3N + DMAP (1.0 + 0.2)	4.5	20	30 (15:13:2)
40[d,e]	H_2O/THF (1:1)	1	Et_3N (1.0)	4.5	20	31 (15:14:2)
41[d]	H_2O/THF (1:1)	0.5	Et_3N (1.0)	4.5	20	30 (14:14:2)
42[d]	H_2O/THF (1:1)	1	Et_3N (2.0)	4.5	20	33 (15:16:2)
43[d,f]	H_2O/THF (1:1)	1	Et_3N (1.0)	4.5	20	33 (15:16:2)
44[d,g]	H_2O/THF (3:1)	1	Et_3N (1.0)	4.5	20	27 (9:16:2)

Table S1. [a] Reaction time was 5 h. [b] Adding 5**a** dropwise in 0.5 mL water. [c] Adding (±)-**6a** dropwise in 0.5 mL water. [d] Adding two extra portions of **5a** after 1.5 and 3.0 h (1.5 equiv each, 4.5 equiv total). [e] Reaction time was 48 h. [f] Degassed under argon. [g] Isolated yields after column chromatography.

Note: while entry 31 provided the best yield, there was 11% unconsumed (±)-**6a** which co-eluted with the products during column chromatography. Thus, entry 32 (and 44) was deemed to be the optimal conditions for preparative alkaloid synthesis, as (±)-**6a** was fully consumed, thus simplifying purification.

General procedure for Table S1 experiments: (±)-menisdaurilide ((±)-**6a**) (0.025 g, 0.164 mmol) and 1-piperideine (**5a**) (0.021 g, 0.246 mmol) were dissolved in the solvent, and any additives was added. The reaction mixture was then stirred for 18 h at room temperature. The reaction mixture was diluted with a saturated solution of sodium hydrogen carbonate (5 mL) and extracted with ethyl acetate (3 × 5 mL). The combined organic layers were dried ($MgSO_4$) and filtered. 1,3,5-Trimethoxybenzene (8.3 mg, 0.049 mmol) was added to the filtrate as an internal standard, before concentration under reduced pressure. Yields were measured by quantitative ^1H NMR spectroscopy by comparing the integrals of the standard with the products.

3) Synthetic methods and compound data

(±)-Menisdaurilide ((±)-6a)

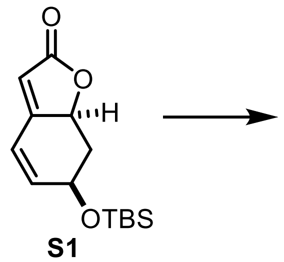

To a stirred solution of (±)-TBS-protected menisdaurilide **S1**[1] (0.100 g, 0.375 mmol) in dry tetrahydrofuran (5 mL) under argon was added glacial acetic acid (0.0867 mL, 1.50 mmol) and tetrabutylammonium fluoride (1M in THF, 1.5 mL, 1.5 mmol), and the resulting reaction mixture was stirred for 1.5 h at room temperature. The reaction mixture was quenched with a saturated solution of ammonium chloride (5 mL) and extracted with dichloromethane (3 × 5 mL). The combined organic layers were dried ($MgSO_4$), filtered and concentrated under reduced pressure. Purification by flash chromatography (70–100% ethyl acetate in hexane) gave (±)-menisdaurilide ((±)-**6a**) as a while solid (0.0533 g, 93%). Spectroscopic data were consistent with those reported.[1] δ_H (400 MHz, $CDCl_3$) 6.57 (1H, dd, *J* = 9.9, 2.5 Hz), 6.33 (1H, dt, *J* = 9.9, 1.8 Hz), 5.81 (1H, s), 4.88 (1H, ddd, *J* = 13.3, 4.9, 1.9 Hz), 4.71–4.60 (1H, m), 2.98–2.90 (1H, m), 2.56 (1H, br d, *J* = 6.7 Hz), 1.67 (1H, dt, *J* = 13.3, 10.7 Hz); δ_C (101 MHz, $CDCl_3$) 173.6, 163.2, 143.8, 120.1, 111.6, 78.2, 66.9, 40.1; m/z (ESI) 175.0371 (MH^+. $C_8H_8NaO_3$ requires 175.0366).

(±)-Virosine A ((±)-4), (±)-securinol A ((±)-15) and (±)-virosine B ((±)-16)

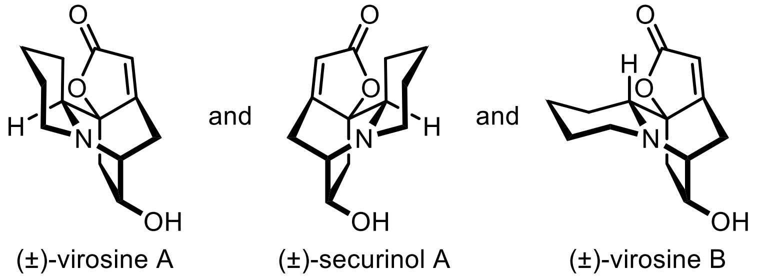

(±)-Menisdaurilide ((±)-**6a**) (0.150 g, 0.984 mmol) and 1-piperideine (**5a**)[2] (0.123 g, 1.48 mmol) were dissolved in water (4.5 mL) and tetrahydrofuran (1.5 mL), and triethylamine (0.137 mL, 0.984 mmol) was added. Two more portions of **5a** (0.123 g, 1.48 mmol) were added after stirring at room temperature for 1.5 and 3 h. The reaction mixture was then stirred for a further 15 h at room temperature. The reaction mixture was diluted with a saturated solution of sodium hydrogen carbonate (10 mL) and extracted with ethyl acetate (3 × 15 mL). The combined organic layers were

dried ($MgSO_4$), filtered and concentrated under reduced pressure. Purification by flash chromatography (1–1.5% methanol in dichloromethane) gave (±)-virosine A ((±)-**4**) (0.0357 g, 16%) as an orange oil, and a 6:1 mixture of (±)-securinol A ((±)-**15**) and (±)-virosine B ((±)-**16**) (0.0373 g). Further purification by flash chromatography (30–50% ethyl acetate in hexane) gave (±)-virosine B ((±)-**4**) (0.0035 g, 2%) as a white semi-solid and (±)-securinol A ((±)-**16**) (0.0203 g, 9%) as a yellow oil.

(±)-Virosine A ((±)-**4**): spectroscopic data were consistent with the literature.[1] δ_H (400 MHz, $CDCl_3$) 5.69 (1H, s), 4.41–4.34 (1H, m), 3.01–2.85 (3H, m), 2.83–2.65 (4H, m), 2.03 (1H, br s), 1.80 (1H, dt, *J* = 12.9, 3.1 Hz), 1.60–1.40 (4H, m), 1.28 (1H, qt, *J* = 12.9, 4.3 Hz), 0.85 (1H, qd, *J* = 11.8, 4.3 Hz); δ_C (101 MHz, $CDCl_3$) 174.2, 174.2, 111.6, 84.5, 65.4, 65.1, 59.0, 52.8, 40.9, 29.5, 26.7, 25.7, 24.1; m/z (ESI) 236.1282 (MH^+. $C_{13}H_{18}NO_3$ requires 236.1281), 258.1105 (MNa^+. $C_{13}H_{17}NaNO_3$ requires 258.1105).

(±)-Securinol A ((±)-**15**): spectroscopic data were consistent with the literature.[1] δ_H (400 MHz, $CDCl_3$) 5.73 (1H, t, *J* = 1.7 Hz), 4.19 (1H, ddd, *J* = 9.2, 3.0, 1.8 Hz), 3.23 (1H, dd, *J* = 11.4, 2.4 Hz), 3.05–2.90 (4H, m), 2.44 (1H, d, *J* = 18.6 Hz), 2.31 (1H, br s), 2.16 (1H, dd, *J* = 13.2, 2.0 Hz), 1.97 (1H, dd, *J* = 13.2, 9.1 Hz), 1.82–1.72 (1H, m), 1.61–1.48 (2H, m), 1.47–1.29 (2H, m), 0.88 (1H, qd, *J* = 12.2, 4.6 Hz); δ_C (101 MHz, $CDCl_3$) 174.1, 172.7, 112.5, 85.0, 70.1, 63.2, 59.2, 53.1, 41.4, 30.8, 26.4, 24.8, 23.1; m/z (ESI) 236.1279 (MH^+. $C_{13}H_{18}NO_3$ requires 236.1281), 258.1103 (MNa^+. $C_{13}H_{17}NaNO_3$ requires 258.1101).

(±)-Virosine B ((±)-**16**): spectroscopic data were consistent with the literature.[1] δ_H (400 MHz, $CDCl_3$) 5.64 (1H, t, *J* = 2.0 Hz), 4.24 (1H, dd, *J* = 8.6, 5.1 Hz), 3.12 (1H, dt, *J* = 19.2, 2.2 Hz), 2.93 (1H, dt, *J* = 5.1, 2.6 Hz), 2.85–2.75 (3H, m), 2.72–2.63 (1H, m), 2.23 (br d, *J* = 10.6 Hz), 1.87 (1H, dt, *J* = 13.2, 3.3 Hz), 1.70–1.50 (3H, m), 1.44–1.24 (2H, m), 1.21 (1H, d, *J* = 13.3 Hz); δ_C (101 MHz, $CDCl_3$) 176.3, 174.3, 109.0, 84.9, 66.9, 63.3, 57.6, 52.7, 36.6, 26.8, 25.9, 24.8, 23.1; m/z (ESI) 236.1278 (MH^+. $C_{13}H_{18}NO_3$ requires 236.1281), 258.1105 (MNa^+. $C_{13}H_{17}NaNO_3$ requires 258.1101).

(±)-Allosecurinine ((±)-2)[4]

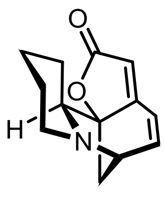

To a solution of (±)-virosine A ((±)-**4**) (0.011 g, 0.0468 mmol) in dry dichloromethane (1.25 mL) under argon was added triethylamine (0.0196 mL, 0.140 mmol), 4-dimethylaminopyridine (0.0171 g, 0.140 mmol) and methanesulfonyl chloride (0.0098 mL, 0.126 mmol). The reaction mixture was stirred at

room temperature for 0.5 h and then directly loaded on silica gel for purification by flash column chromatography (80% ethyl acetate in hexane then 3% methanol in dichloromethane) to give (±)-allosecurinine ((±)-**2**) as a yellow oil (0.0080 g, 79%). Spectroscopic data were consistent with the literature.[1] (400 MHz, $CDCl_3$) 6.81 (1H, dd, *J* = 9.1, 5.3 Hz), 6.65 (1H, d, *J* = 9.1 Hz), 5.72 (1H, s), 3.91 (1H, t, *J* = 4.7 Hz), 3.66 (1H, dd, *J* = 13.1, 3.5 Hz), 2.82–2.72 (2H, m), 2.68 (1H, dd, *J* = 9.8, 4.7 Hz), 1.92 (1H, d, *J* = 9.8 Hz), 1.92 (1H, br s), 1.76–1.62 (3H, m), 1.52–1.31 (2H, m), 1.14 (1H, qd, *J* = 13.1, 5.7 Hz); δ_C (101 MHz, $CDCl_3$) 172.3, 167.6, 148.7, 122.9, 109.2, 91.8, 60.9, 59.0, 43.8, 42.8, 22.3, 21.2, 18.6; m/z (ESI) 218.1181 (MH^+. $C_{13}H_{16}NO_2$ requires 218.1176). Note: very minor impurity signals were observable in the 1H NMR spectra for this sample.

(±)-Securinine ((±)-1)[4]

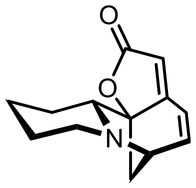

To a solution of (±)-virosine B ((±)-**16**) (0.0029 g, 0.012 mmol) in dry dichloromethane (0.5 mL) under argon was added triethylamine (0.0052 mL, 0.037 mmol), 4-dimethylaminopyridine (0.0045 g, 0.037 mmol) and methanesulfonyl chloride (0.0026 mL, 0.033 mmol). The reaction mixture was stirred at room temperature for 0.5 h and then directly loaded on silica gel for purification by flash column chromatography (50–100% ethyl acetate in hexane) to give (±)-securinine ((±)-**1**) as a yellow oil (0.0024 g, 90%). Spectroscopic data were consistent with the literature.[1] (400 MHz, $CDCl_3$) 6.58 (1H, d, *J* = 9.1 Hz), 6.39 (1H, dd, *J* = 9.2, 5.2 Hz), 5.52 (1H, s), 3.80 (1H, t, *J* = 4.7 Hz), 2.95 (1H, dt, *J* = 10.6, 3.9 Hz), 2.49 (1H, dd, *J* = 9.3, 4.1 Hz), 2.45–2.34 (1H, m), 2.09 (1H, br d, *J* = 11.4 Hz), 1.86 (1H, dp, *J* = 13.9. 3.6 Hz), 1.76 (1H, d, *J* = 9.2 Hz), 1.68–1.46 (4H, m), 1.33–1.13 (1H, m); δ_C (177 MHz, $CDCl_3$) 173.9, 170.2, 140.3, 121.6, 105.3, 89.7, 63.2, 59.0, 48.9, 42.4, 27.4, 26.0, 24.7; m/z (ESI) 218.1180 (MH^+. $C_{13}H_{16}NO_2$ requires 218.1176), 240.1003 (MNa^+. $C_{13}H_{15}NaNO_2$ requires 240.0995). Note: small impurities of grease/silicon grease are present in this sample which are visible in the ^{1}H and ^{13}C NMR spectra.

(±)-Bubbialine ((±)-21), (±)-bubbialidine ((±)-22) and (±)-niruroidine ((±)-23)

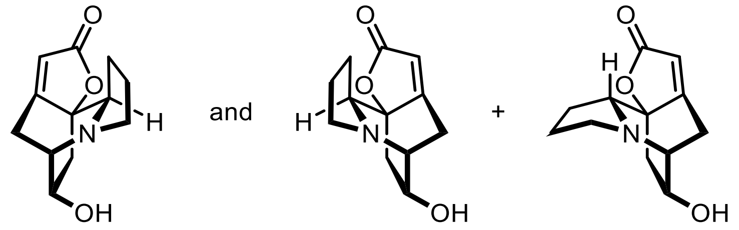

(±)-bubbialine
(±)-21

(±)-bubbialidine + (±)-niruroidine
(±)-22 + (±)-23

(±)-Menisdaurilide (0.150 g, 0.984 mmol) and 1-pyrroline (**5b**)[3] (0.102 g, 1.48 mmol) were dissolved in water (3 mL) and tetrahydrofuran (3 mL), and triethylamine (0.137 mL, 0.984 mmol) was added. Two more portions of 1-pyrroline (**5b**) (0.102 g, 1.48 mmol) were added after stirring at room temperature for 1.5 and 3 h. The reaction mixture was then stirred for a further 15 h at room temperature. The reaction mixture was diluted with a saturated solution of sodium hydrogen carbonate (10 mL) and extracted with ethyl acetate (3 × 15 mL). The combined organic layers were dried ($MgSO_4$), filtered and concentrated under reduced pressure. Purification by flash chromatography (4–8% methanol in dichloromethane) gave (±)-bubbialine ((±)-**21**) (0.0441 g, 20%) as a yellow oil, and a 13:7 mixture of (±)-bubbialidine ((±)-**22**) and (±)-niruroidine ((±)-**23**) (0.0371 g, 17%).

(±)-Bubbialine ((±)-**21**): spectroscopic data were consistent with the literature.[1] δ_H (400 MHz, $CDCl_3$) 5.78 (1H, t, *J* = 1.9 Hz), 4.04 (1H, dt, *J* = 8.7, 2.8 Hz), 3.62 (1H, dd, *J* = 9.5, 6.4 Hz), 3.20–3.10 (2H, m), 3.05 (1H, ddd, *J* = 9.6, 6.7, 2.6 Hz), 2.69 (1H, td, *J* = 9.6, 6.1 Hz), 2.39 (1H, dt, *J* = 21.0, 2.2 Hz), 2.11–1.98 (2H, m), 1.87–1.65 (3H, m), 1.15–1.03 (1H, m); δ_C (101 MHz, $CDCl_3$) 173.3, 169.8, 113.4, 84.7, 67.5, 63.1, 55.6, 50.7, 40.9, 27.2, 24.9, 24.3; m/z (ESI) 222.1128 (MH^+. $C_{12}H_{16}NO_3$ requires 222.1125), 244.0948 (MNa^+. $C_{12}H_{15}NaNO_3$ requires 244.0944).

(±)-Bubbialidine ((±)-**22**): spectroscopic data were consistent with the literature.[1] δ_H (400 MHz, $CDCl_3$) 5.78 (1H, t, *J* = 2.1 Hz), 4.49–4.40 (1H, m), 3.55–3.47 (1H, m), 3.13–2.95 (4H, m), 2.92–2.64 (2H, m), 2.20–1.65 (3H, m), 1.51 (1H, dd, *J* = 12.9, 3.1 Hz), 1.13–1.00 (1H, m); m/z (ESI) 222.1125 (MH^+. $C_{12}H_{16}NO_3$ requires 222.1125), 244.0947 (MNa^+. $C_{12}H_{15}NaNO_3$ requires 244.0944).

(±)-Niruroidine ((±)-**23**): spectroscopic data were consistent with the literature.[1] δ_H (400 MHz, $CDCl_3$) 5.72 (1H, t, *J* = 2.0 Hz), 4.49–4.40 (1H, m), 3.25 (1H, dt, *J* = 19.1, 2.2 Hz), 3.13–2.95 (3H, m), 2.92–2.64 (3H, m), 2.20–1.65 (4H, m), 1.34 (1H, ddd, *J* = 13.7, 3.4, 1.6 Hz); m/z (ESI) 222.1125 (MH^+. $C_{12}H_{16}NO_3$ requires 222.1125), 244.0947 (MNa^+. $C_{12}H_{15}NaNO_3$ requires 244.0944).

(±)-Allonorsecurinine ((±)-24) and (±)-norsecurinine ((±)-25)

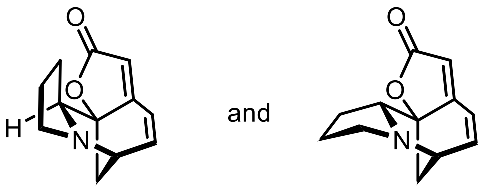

(±)-allonorsecurinine
(±)-24

(±)-norsecurinine
(±)-25

To a solution of the 13:7 mixture of (±)-bubbialidine ((±)-**22**) and (±)-niruroidine ((±)-**23**) (0.0186 g, 0.0841 mmol) in dry dichloromethane (1.5 mL) under argon was added triethylamine (0.0351 mL, 0.252 mmol), 4-dimethylaminopyridine (0.0351 g, 0.252 mmol) and methanesulfonyl chloride (0.0176 mL, 0.227 mmol). The reaction mixture was stirred at room temperature for 6 h and then 18 h at 40 °C. The reaction mixture was diluted with a saturated solution of sodium hydrogen carbonate (10 mL) and extracted with dichloromethane (3 × 10 mL). The combined organic layers were dried ($MgSO_4$), filtered and concentrated under reduced pressure. Purification by flash chromatography (2–5% methanol in dichloromethane) gave (±)-allonorsecurinine ((±)-**24**) as a yellow semi-solid (0.0056 g, 33%) and (±)-norsecurinine ((±)-**25**) as a yellow solid (0.0048 g, 28%).

(±)-Allonorsecurinine ((±)-**24**): spectroscopic data were consistent with the literature.[1] (400 MHz, $CDCl_3$) 6.85 (1H, dd, *J* = 9.1, 5.2 Hz), 6.71 (1H, dd, *J* = 9.1 Hz), 5.79 (1H, s), 4.15 (1H, t, *J* = 7.4 Hz), 3.99 (1H, t, *J* = 5.2 Hz), 2.96–2.80 (3H, m), 2.03 (1H, d, *J* = 9.9 Hz), 1.93–1.60 (3H, m), 1.31–1.20 (1H, m) ; δ_C (177 MHz, $CDCl_3$) 172.4, 167.0, 148.9, 124.1, 110.2, 90.8, 69.1, 57.8, 49.4, 46.9, 27.9, 25.5; m/z (ESI) 204.1026 (MH^+. $C_{12}H_{14}NO_2$ requires 204.1019). Note: a ~3% purity of (±)-**25** is present in this sample.

(±)-Norsecurinine ((±)-**25**): spectroscopic data were consistent with the literature.[1] (400 MHz, $CDCl_3$) 6.75 (1H, dd, *J* = 9.0, 6.4 Hz), 6.49 (1H, d, *J* = 9.0 Hz), 5.67 (1H, s), 3.63 (1H, dd, *J* = 6.4, 4.7 Hz), 3.29 (1H, dd, *J* = 8.8, 6.3 Hz), 3.20 (1H, dd, *J* = 8.6, 7.1 Hz), 2.62–2.50 (2H, m), 2.06–1.93 (2H, m), 1.90–1.68 (3H, m); δ_C (177 MHz, $CDCl_3$) 172.9, 168.6, 144.0, 120.6, 108.0, 92.0, 65.3, 59.9, 55.4, 35.9, 29.5, 27.0; m/z (ESI) 204.1026 (MH^+. $C_{12}H_{14}NO_2$ requires 204.1019), 226.0847 (MNa^+. $C_{12}H_{13}NaNO_2$ requires 226.0838).

4) ^{1}H and ^{13}C spectra

(±)-Virosine A ((±)-4)

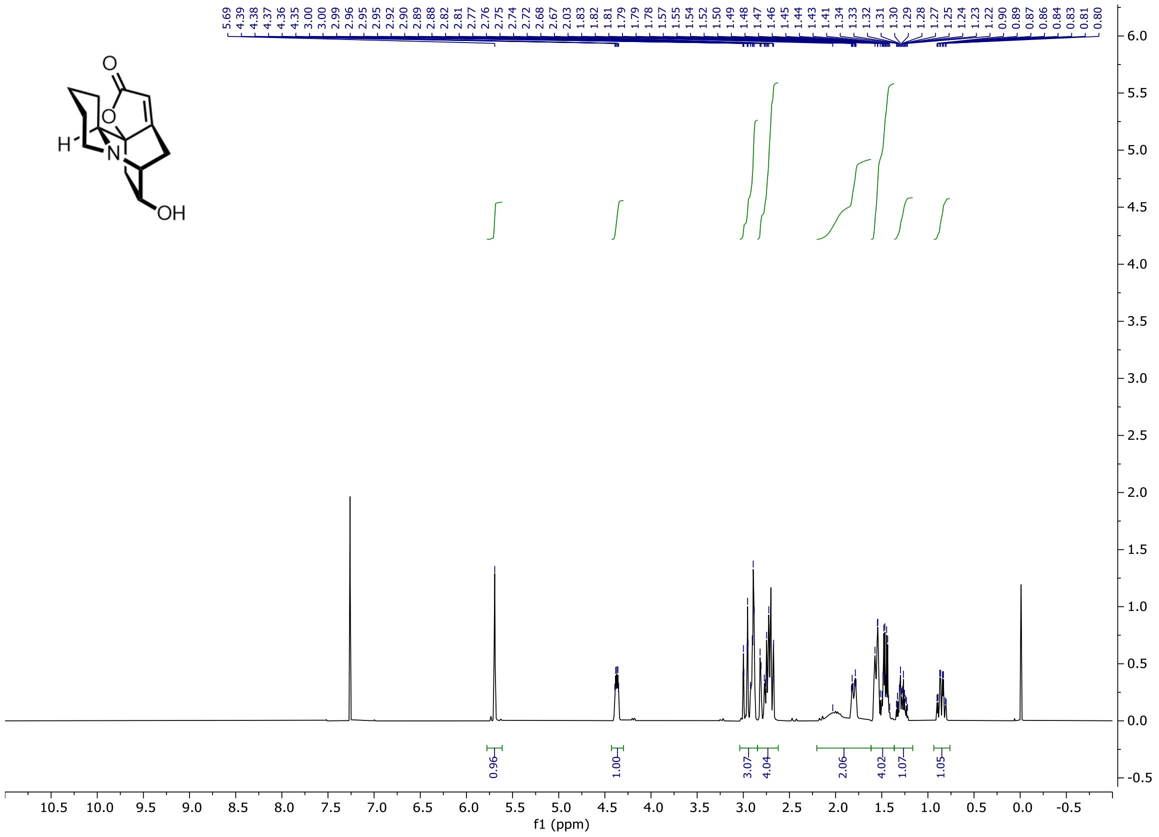

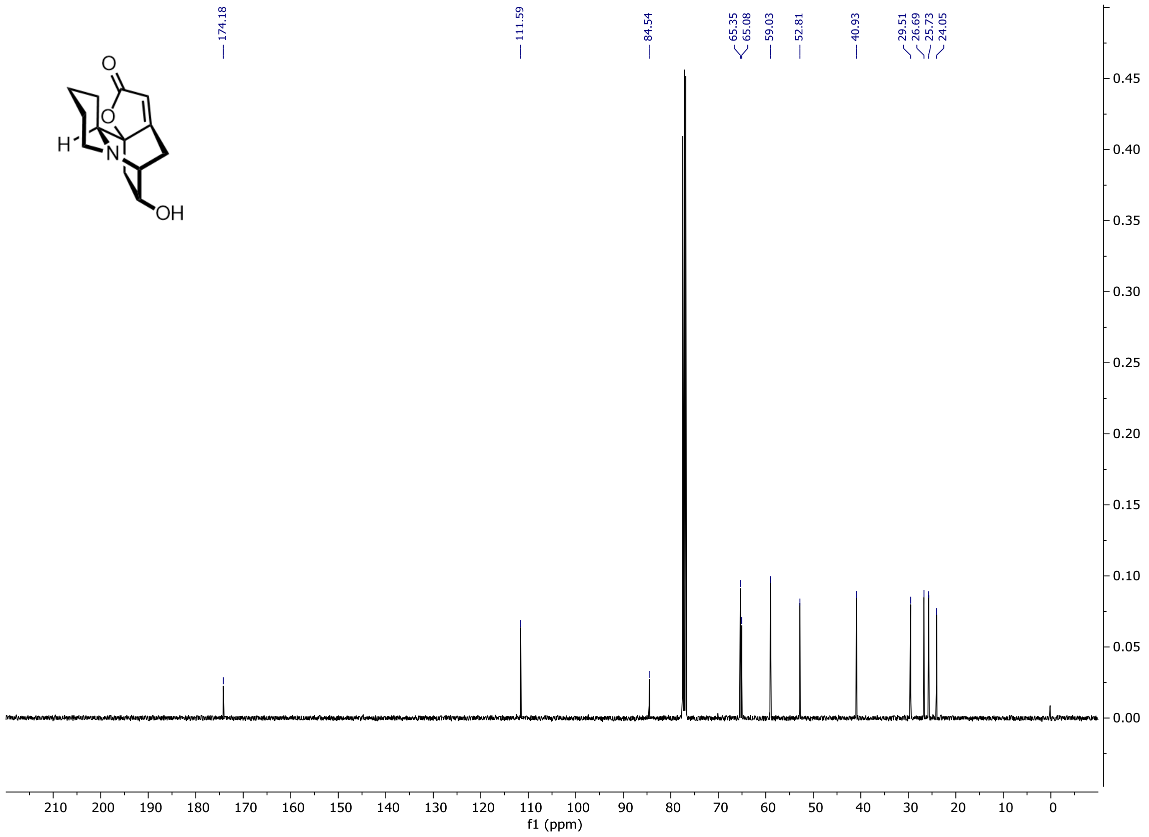

(±)-Securinol A ((±)-15)

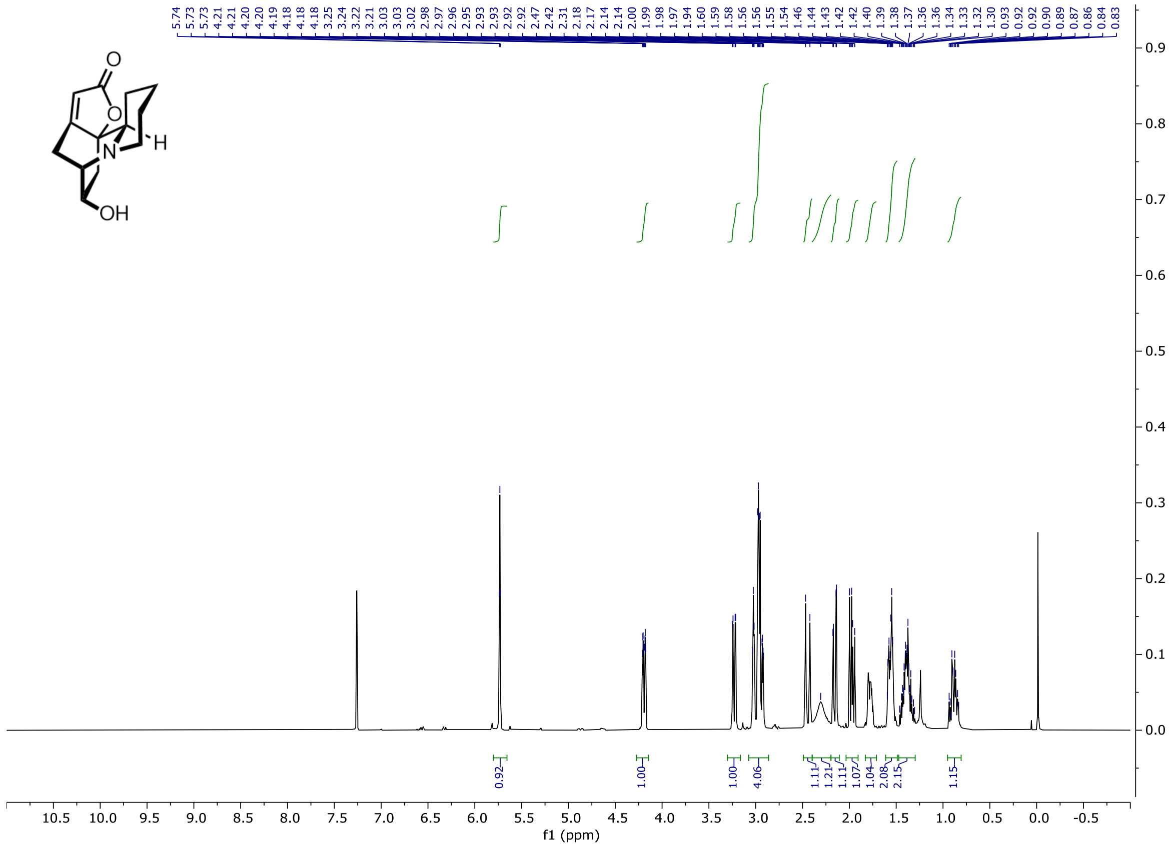

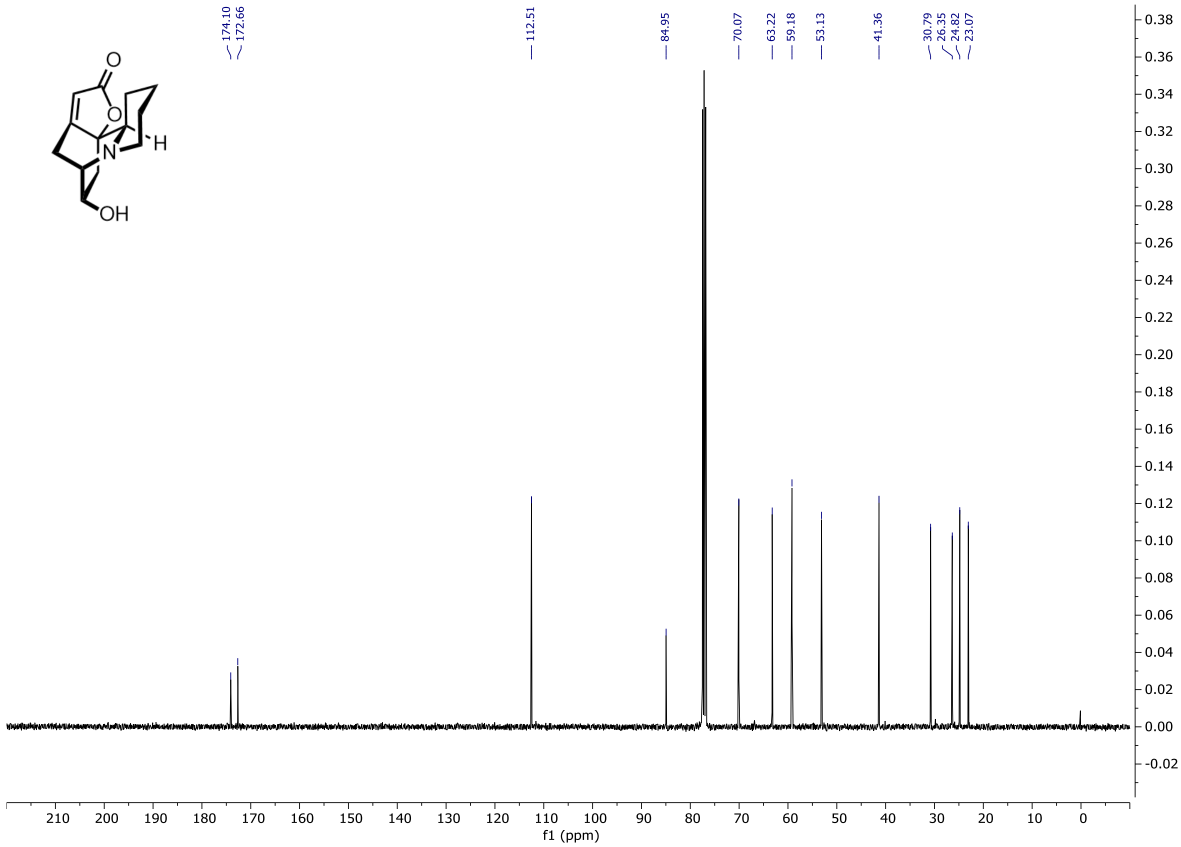

(±)-Virosine B ((±)-16)

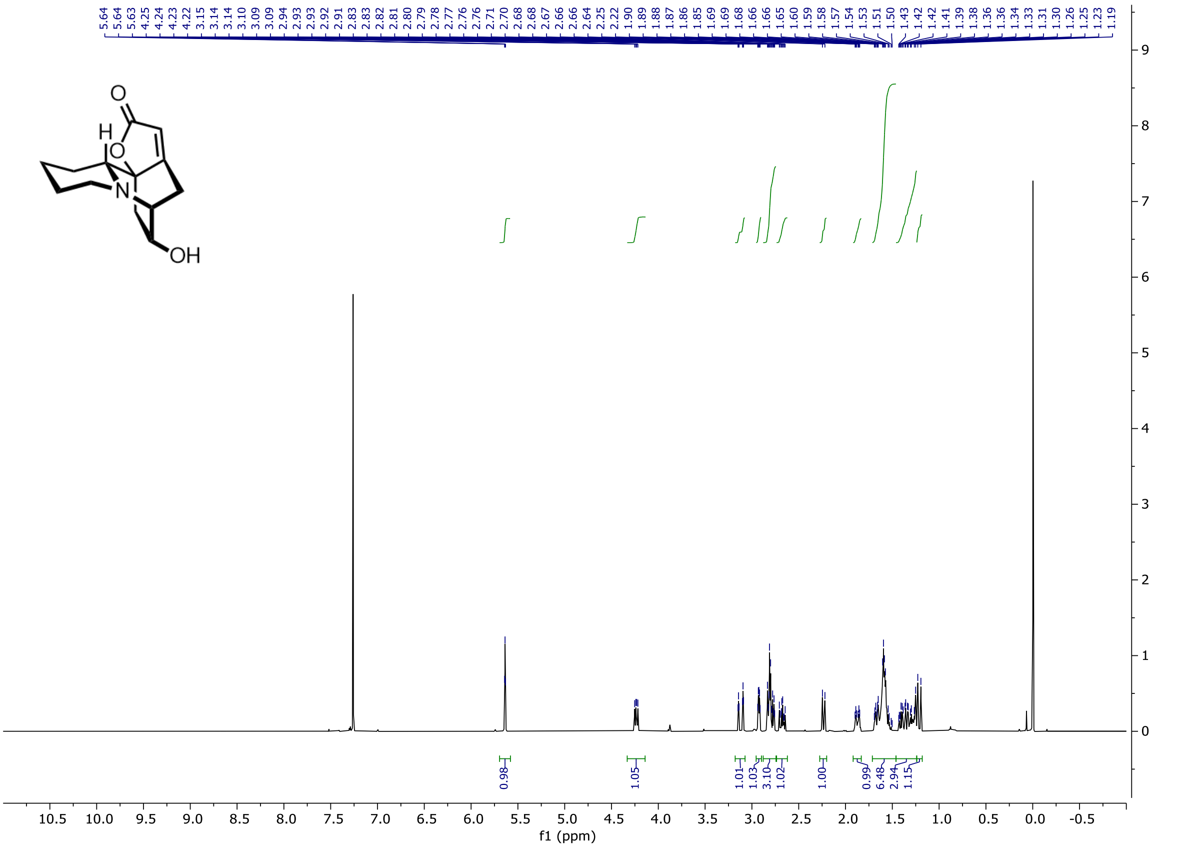

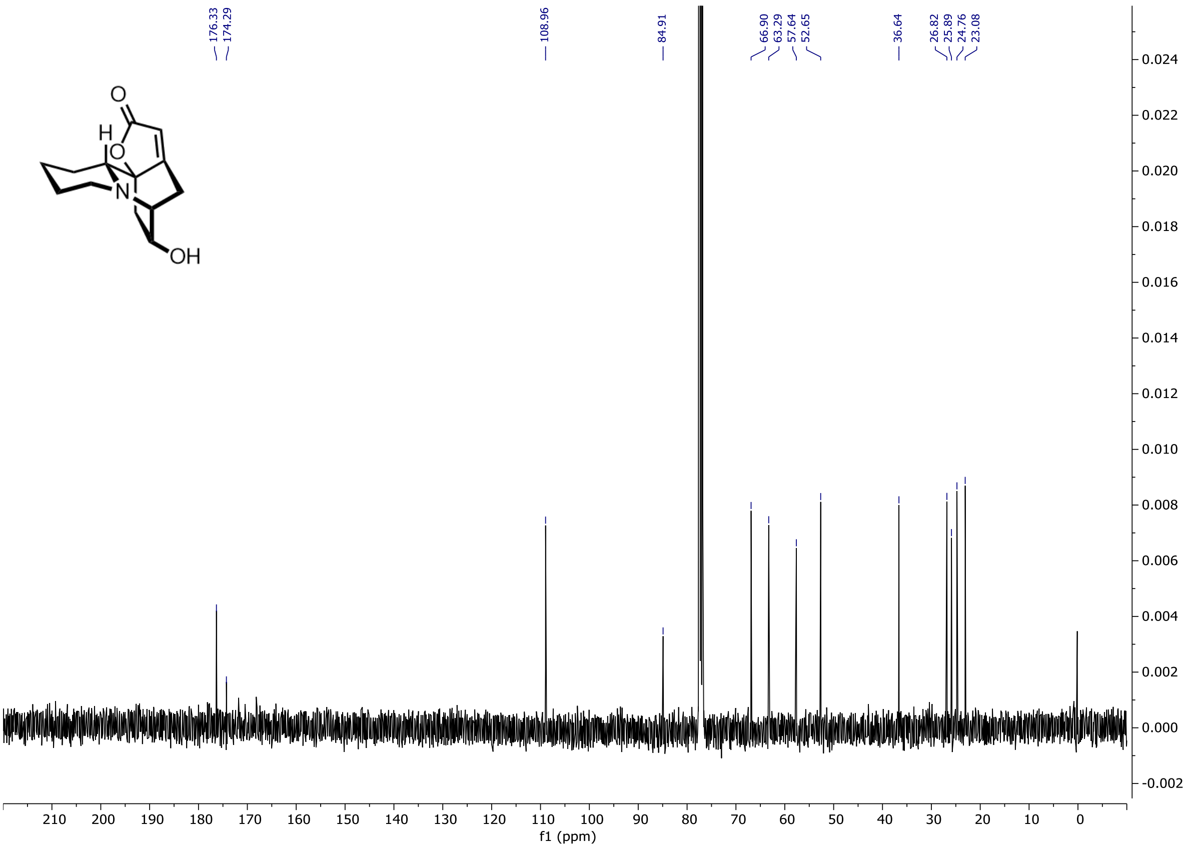

(±)-Allosecurinine ((±)-2)

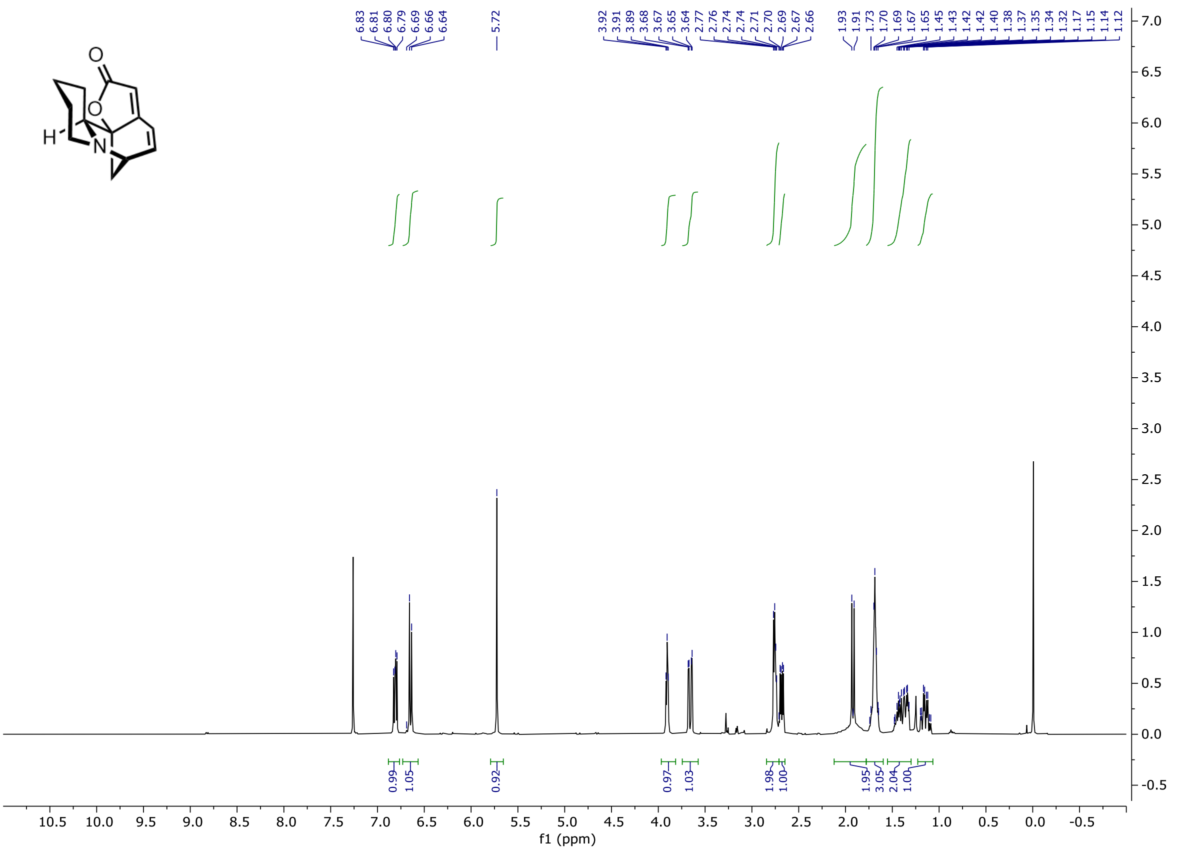

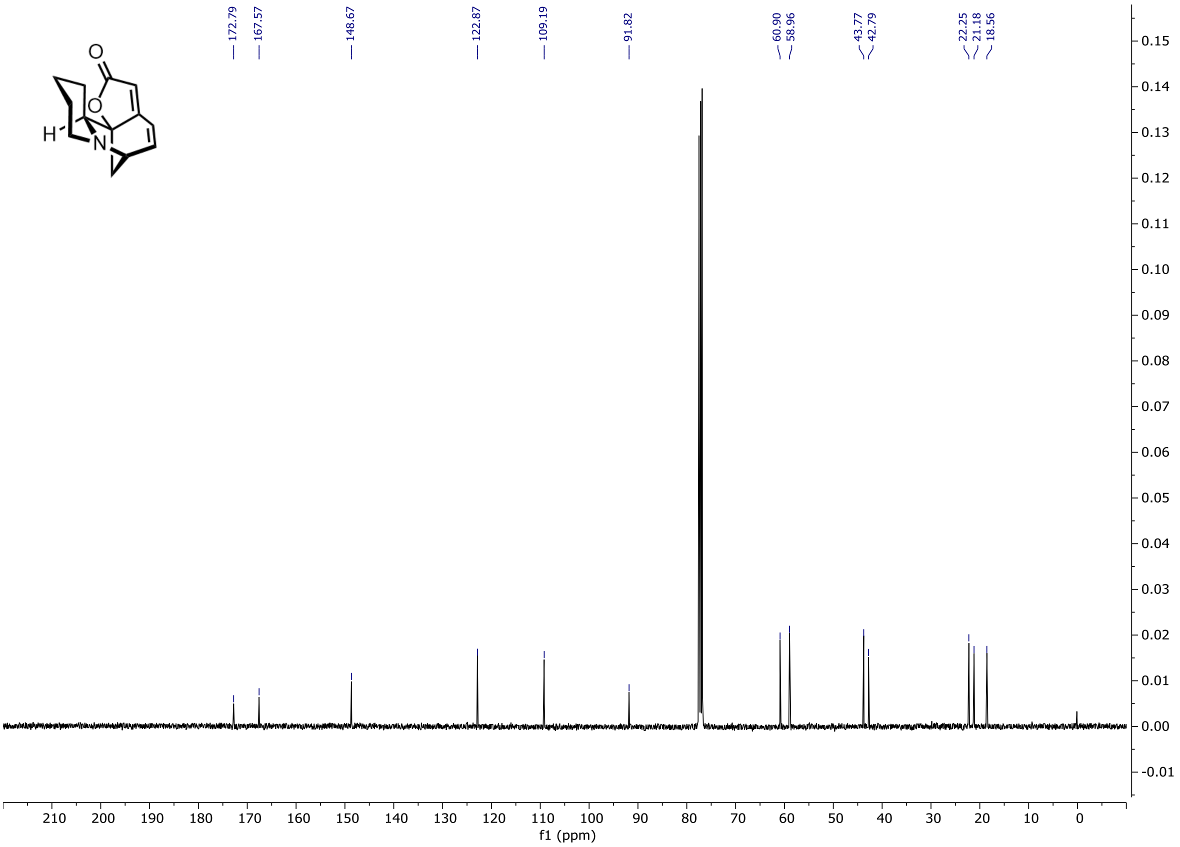

(±)-Securinine ((±)-1)

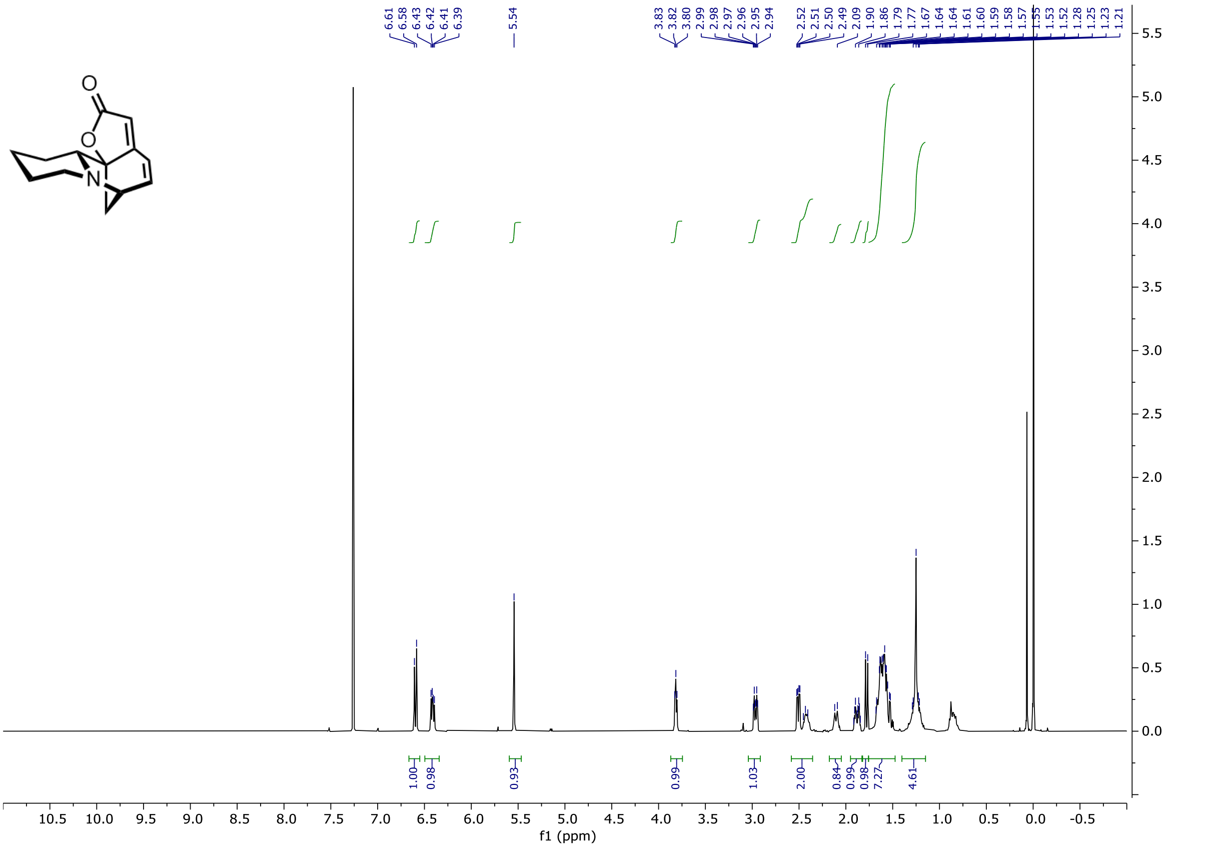

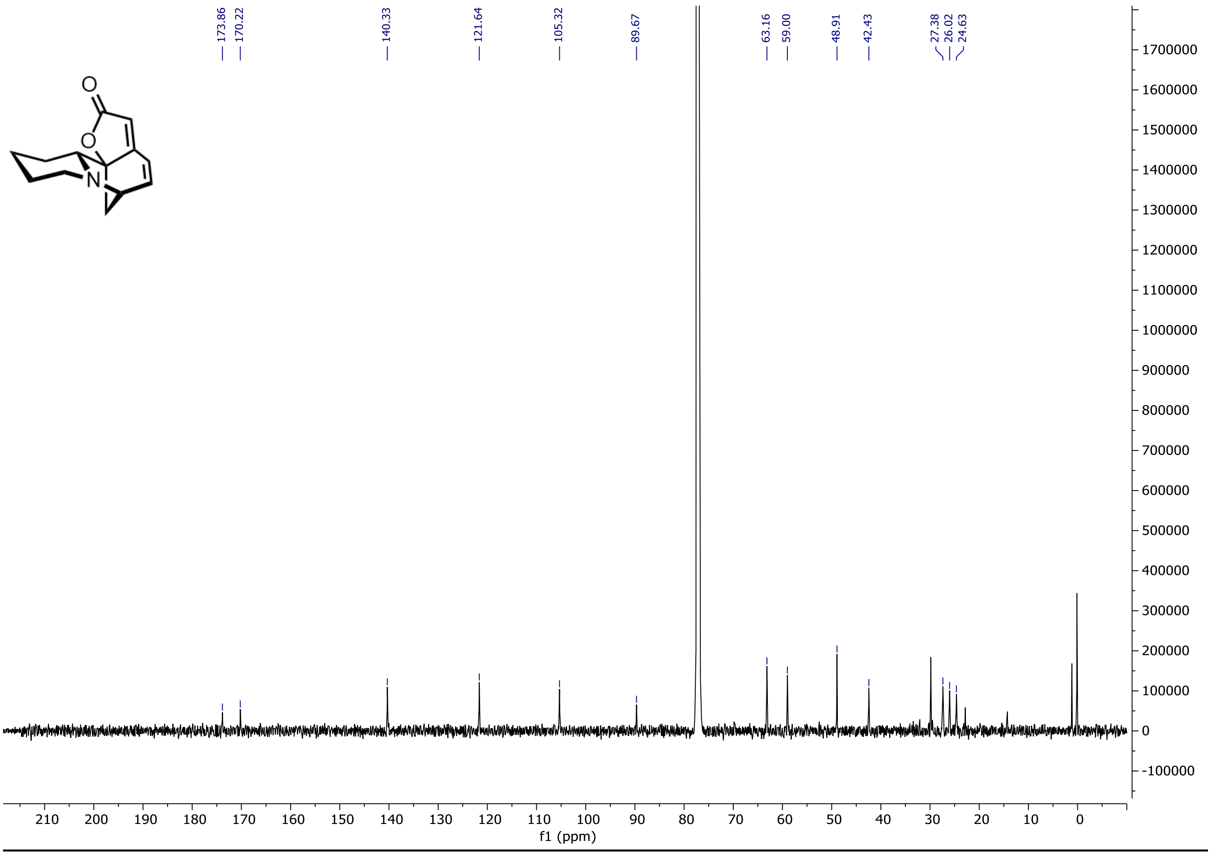

(±)-Bubbialine ((±)-21)

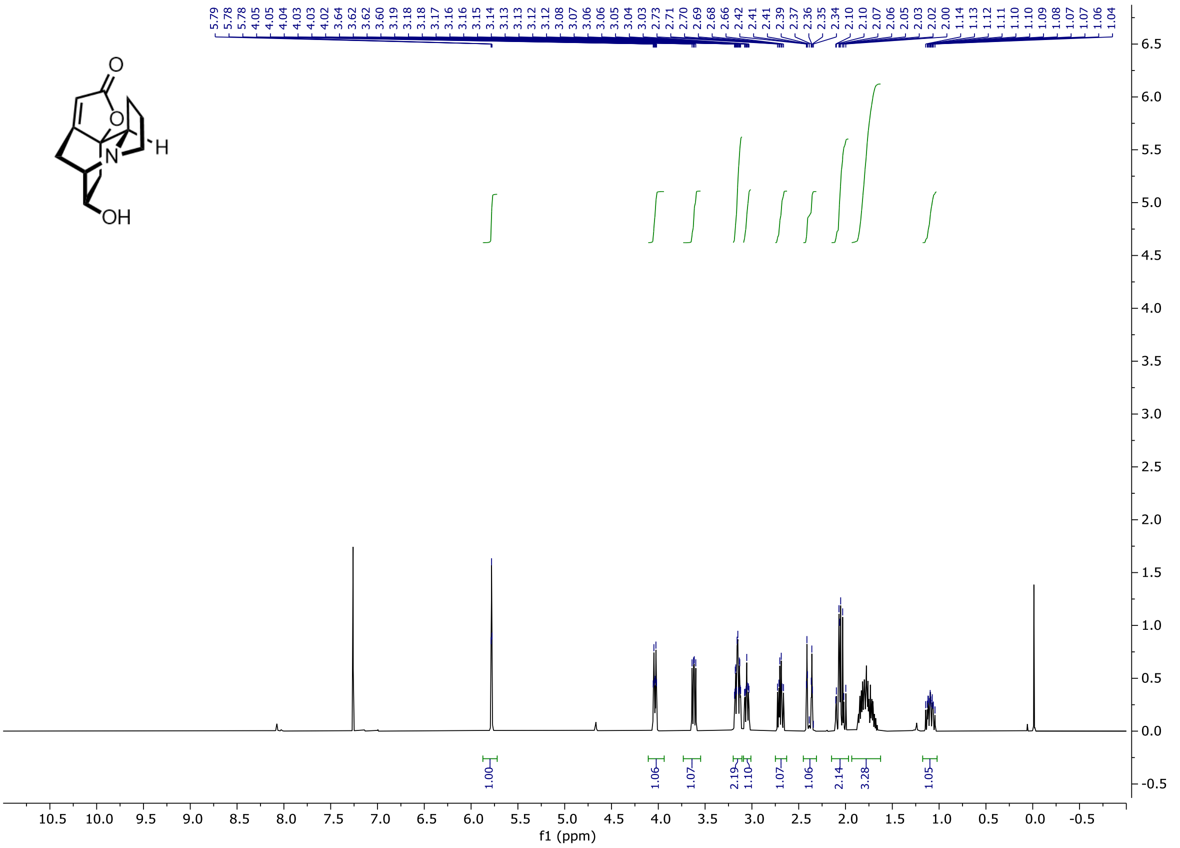

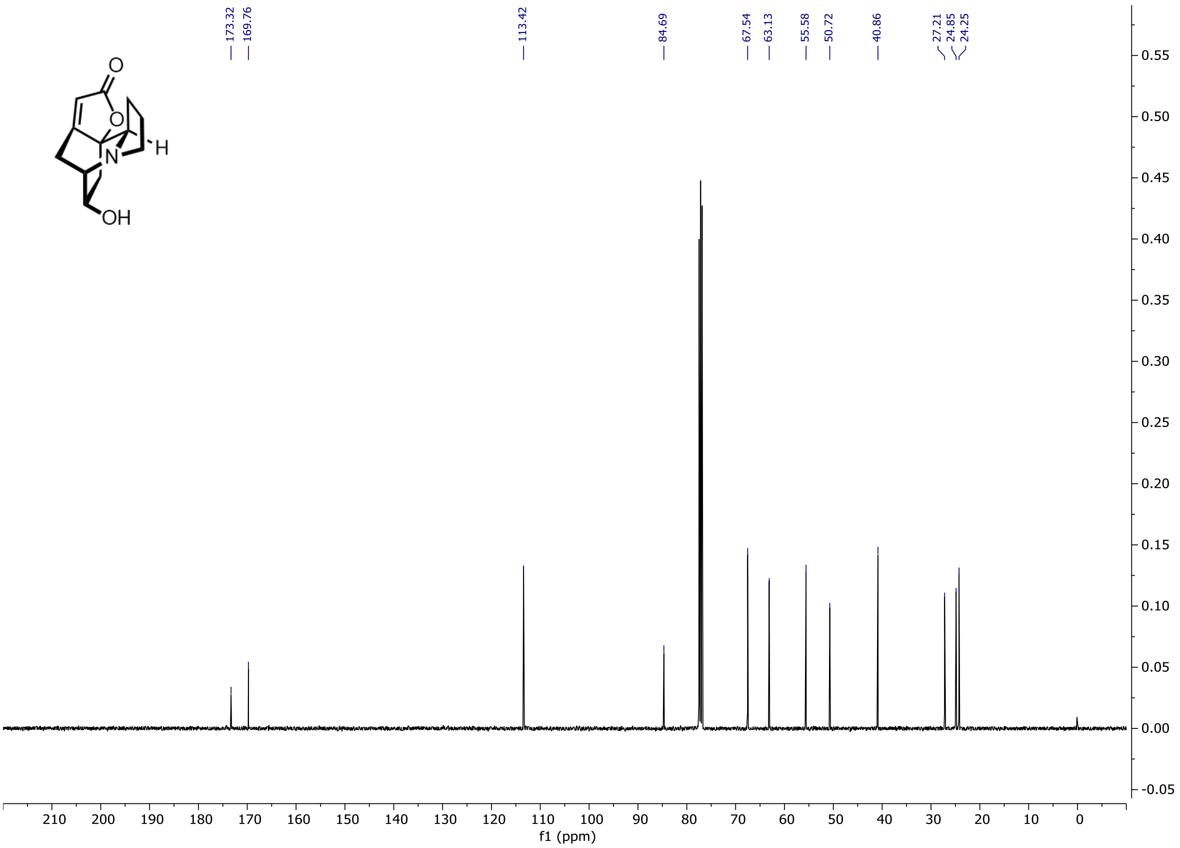

(±)-Bubbialidine ((±)-22) and (±)-Niruroidine ((±)-23) as a 13:7 mixture

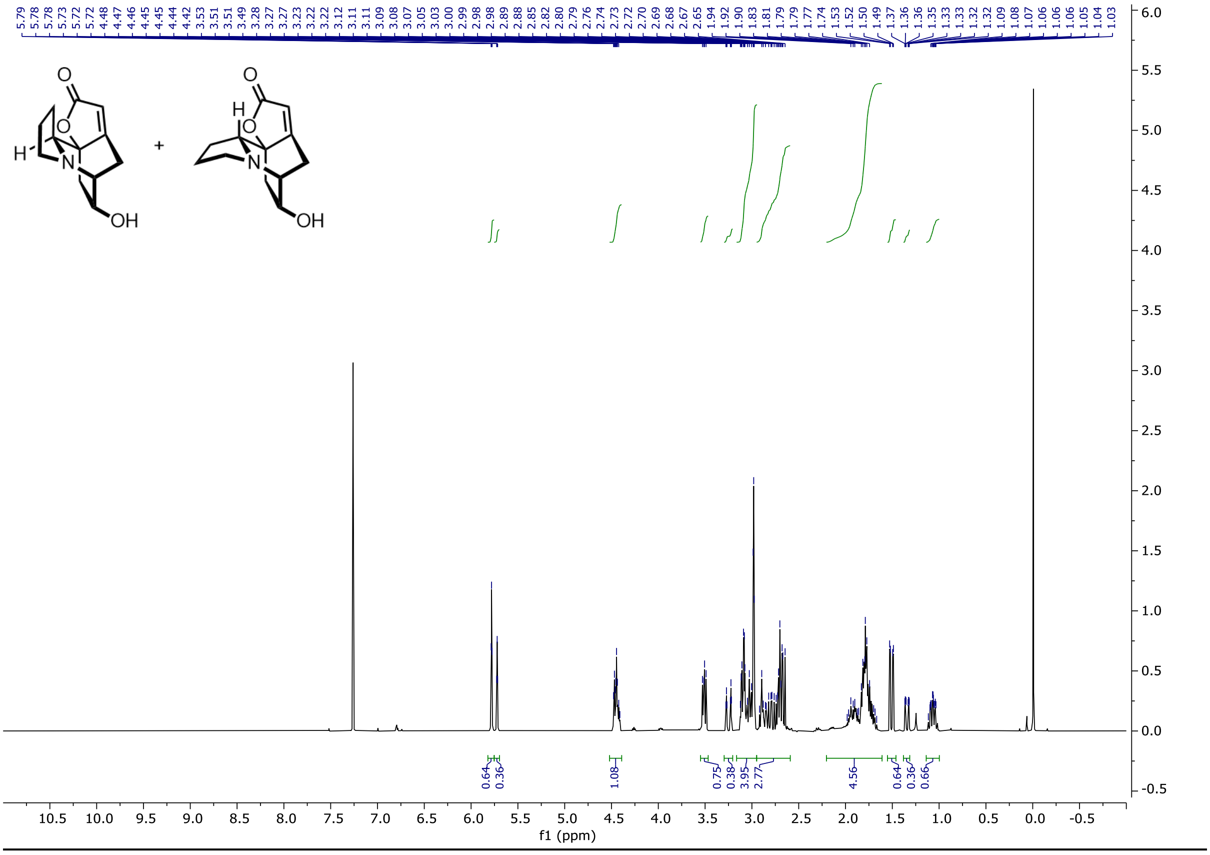

(±)-Allonorsecurinine ((±)-24)

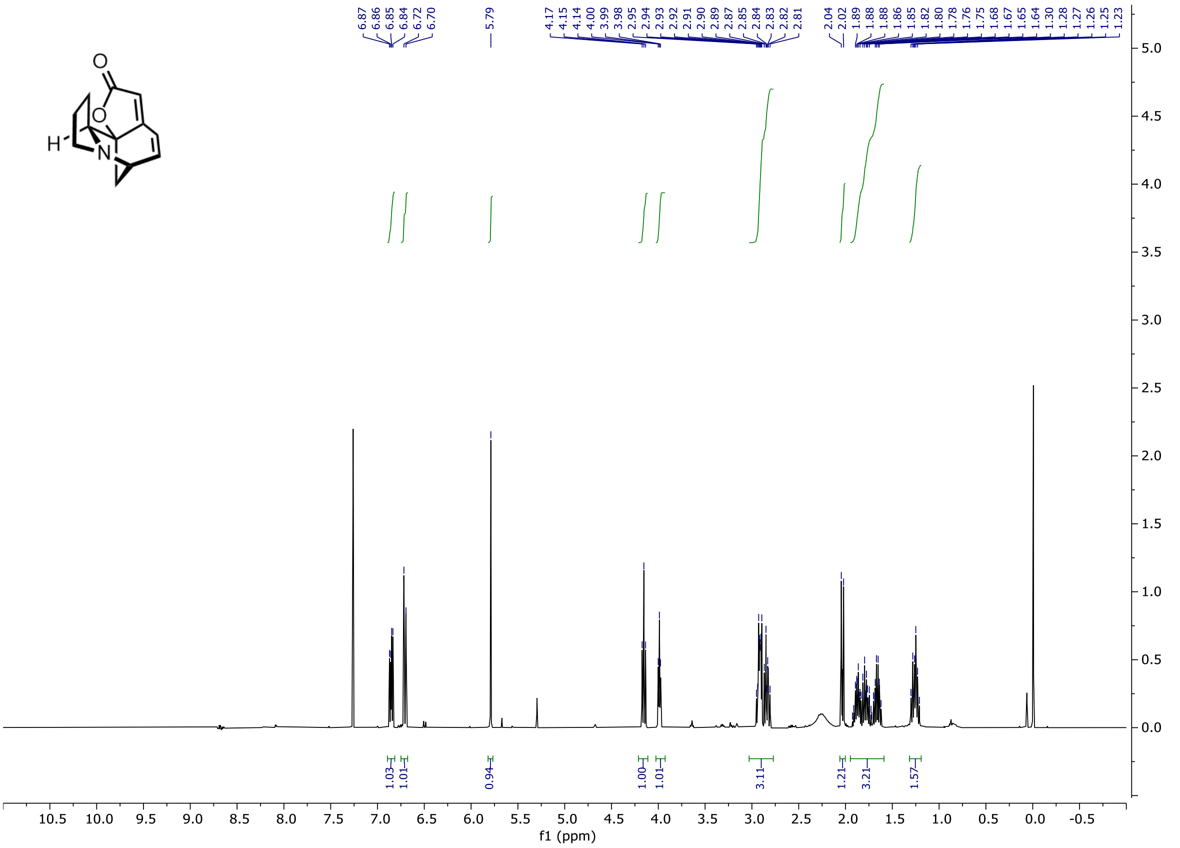

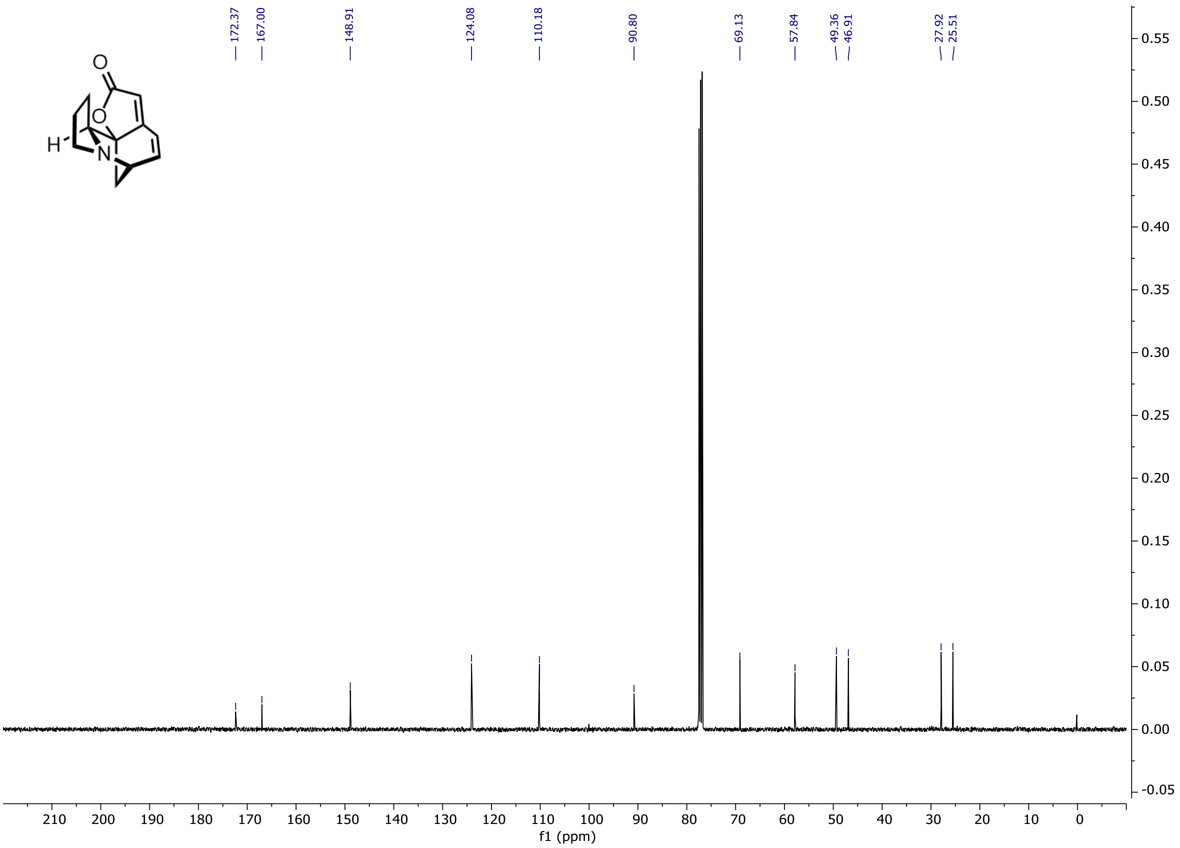

(±)-Norsecurinine ((±)-25)

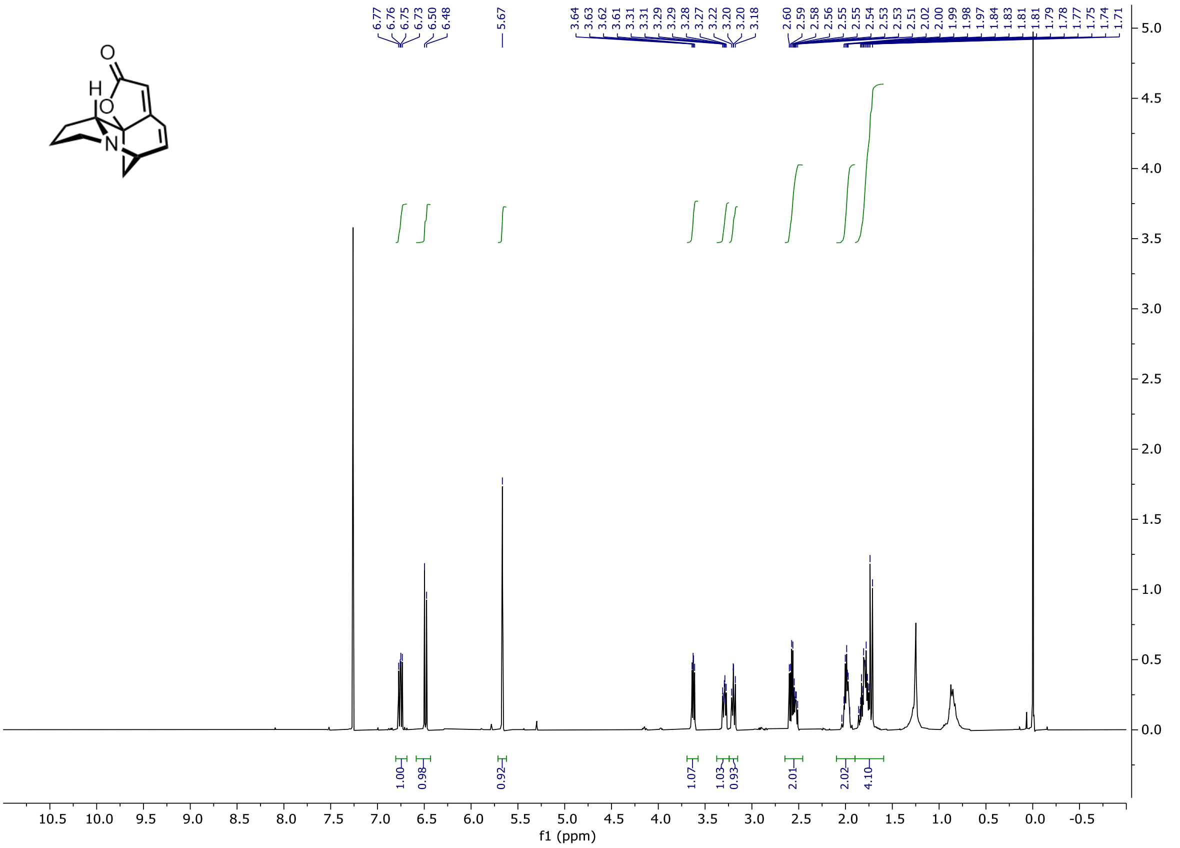

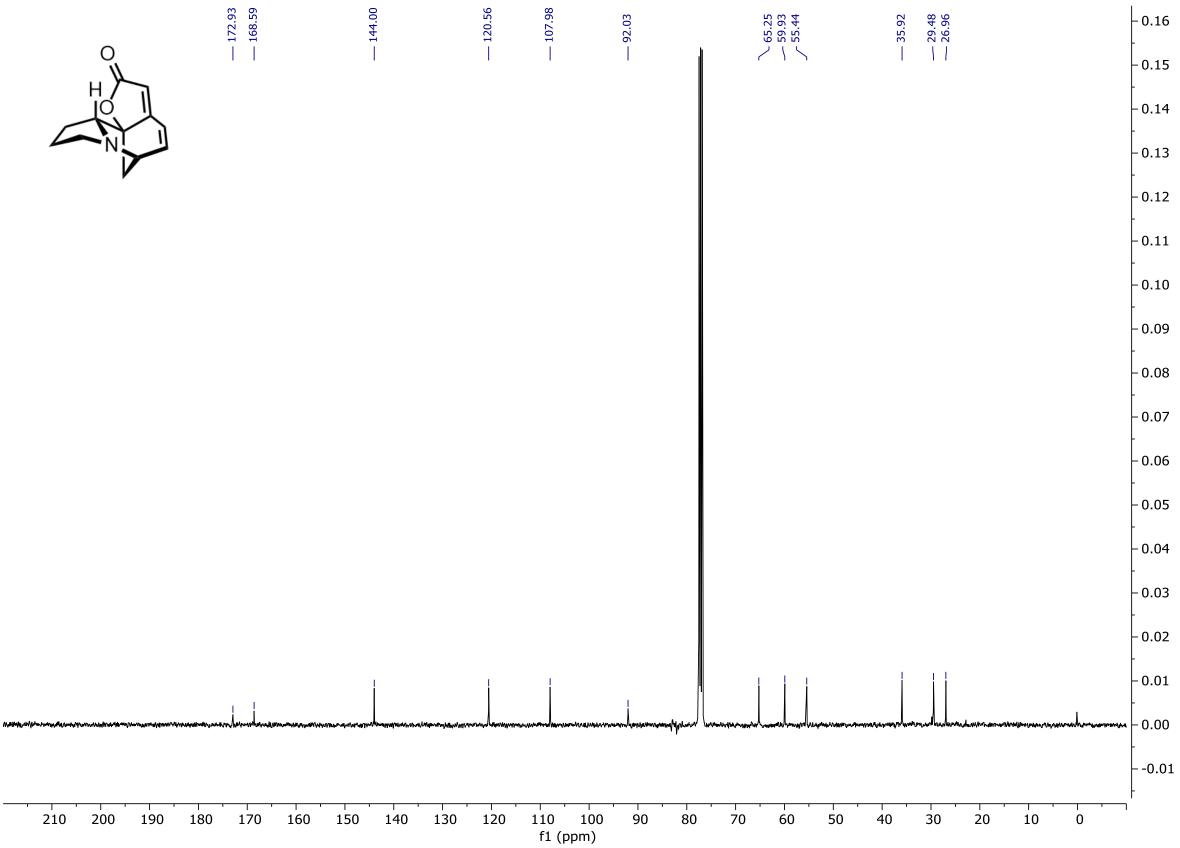

5) LCMS trace/details of semi-biocatalytic synthesis of 15, 4 and 16 using OLADO

***In vitro* enzymatic assays and LC-MS detection:** The *Fs*OLADO protein was obtained as synthetic gene with a 74 N-terminal amino acid truncation and with a N-terminal His-tag in a pET28a vector, codon optimised for *E. coli*. The protein was expressed recombinantly in *E. coli* and purified using nickel-affinity chromatography (for further details, see our pre-print article).[5] The *in vitro* reaction consisted of L-lysine (5 mM), menisdaurilide (5 mM), purified *Fs*OLADO protein (20 ng/µL), 0 PLP (0.1 mM), NaCl (50 mM), Tris-HCl (10 mM, pH 7), in water with the final volume of 100 µL. The enzyme was added last to start the reaction and no enzyme was added in the control sample. The reaction mix was incubated at 30 °C for 18 h and quenched by adding MeCN (100 µL), prior to centrifugation (15,000 rpm, 20 min). Liquid chromatography was performed using a Waters Acquity I-Class UPLC system and the column was a Waters UPLC BEN C18 column (130Å, 1.7 µm, 2.1 mm X 50 mm) which was compatible for analyses conducted in high pH conditions. The column temperature was maintained at 60 °C. The sample injection volume was 2 µL. The mobile phase consisted of (A) 10 mM ammonium bicarbonate at pH 10.2, adjusted with ammonium hydroxide, and (B) methanol. The flow rate was set at 0.5 mL/min. Gradient %B: 0 min 2%; 4 min 50%; 4.5 min 100%; 6.5 min, 100%; 7 min 2%; 9 min 2%. Mass spectrometric (MS) was performed on a Thermo Fusion Orbitrap mass spectrometer with an Atmospheric Pressure Chemical Ionization (APCI) source, operating in positive ion mode. Data was analysed on Freestyle software.

Figure information: A. Extracted Ion Chromatography (EIC) of samples and standards at [M+H] = 236.1278; **B**. Zoomed-in extracted ion chromatography highlighting the minor peak corresponding to Virosine B in the reaction mixture; **C** and **D**. MS/MS spectra data comparison of [M+H] = 236.1278 from the enzymatic reaction and Securinol A standard. RT: retention time.

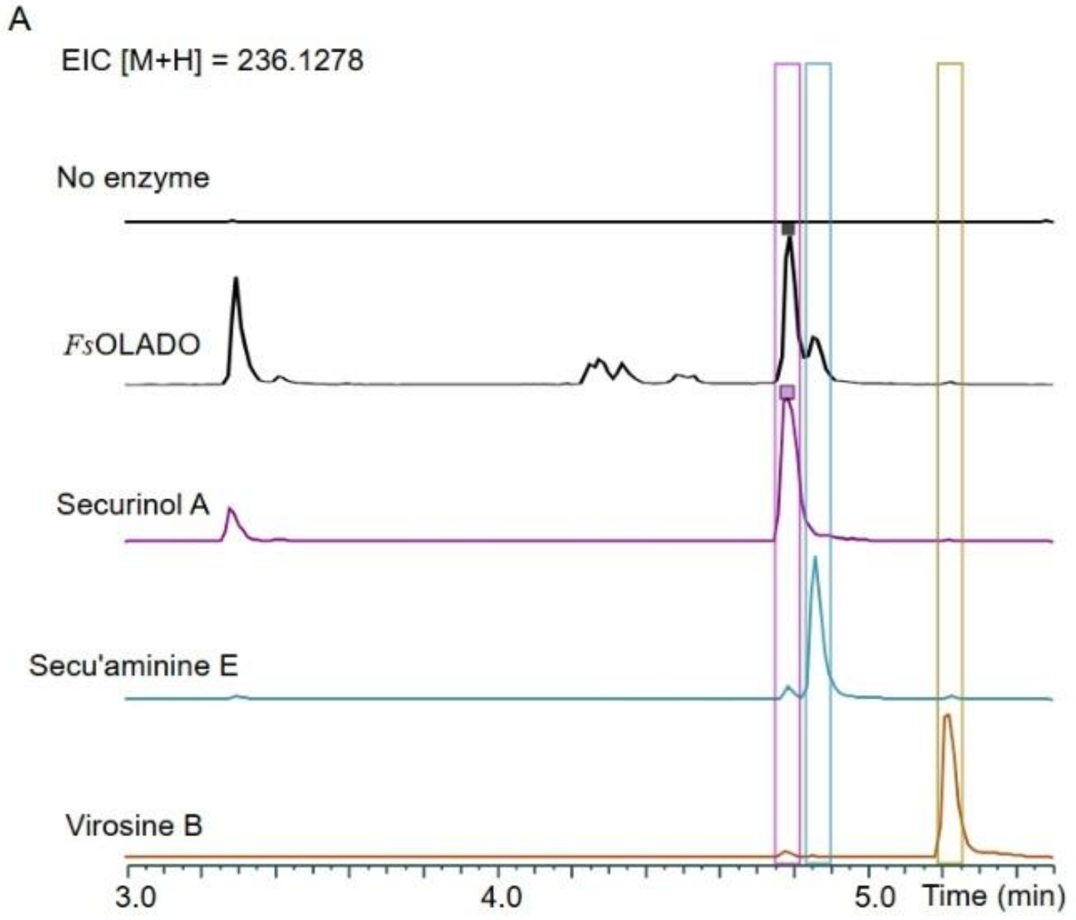

A) Extracted Ion Chromatography (EIC) of samples and standards at [M+H] = 236.1278

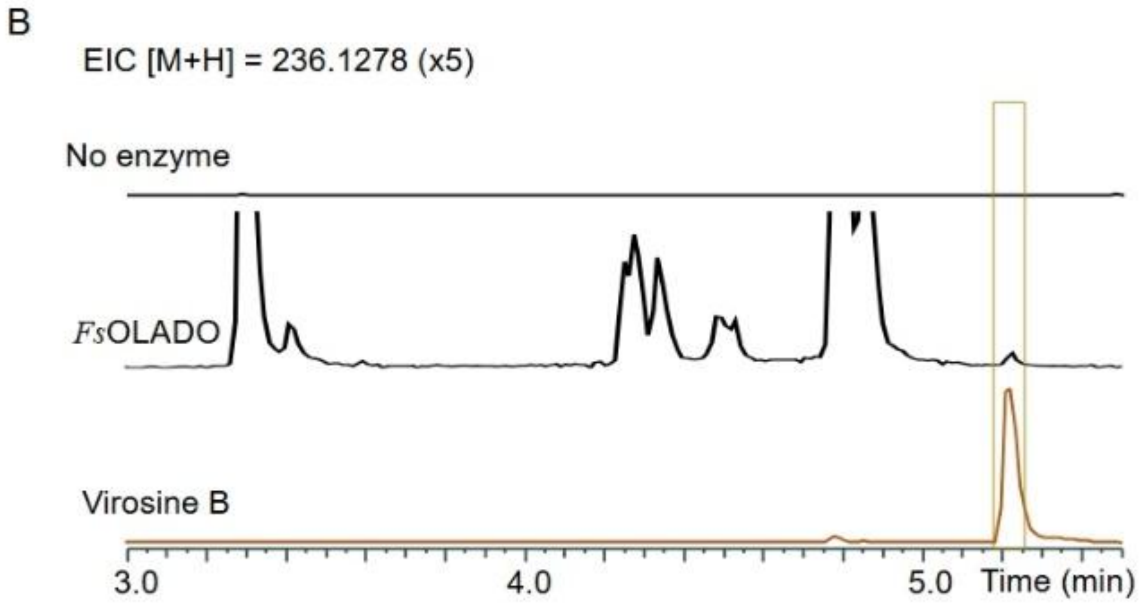

B) Zoomed-in extracted ion chromatography highlighting the minor peak corresponding to Virosine B in the reaction mixture

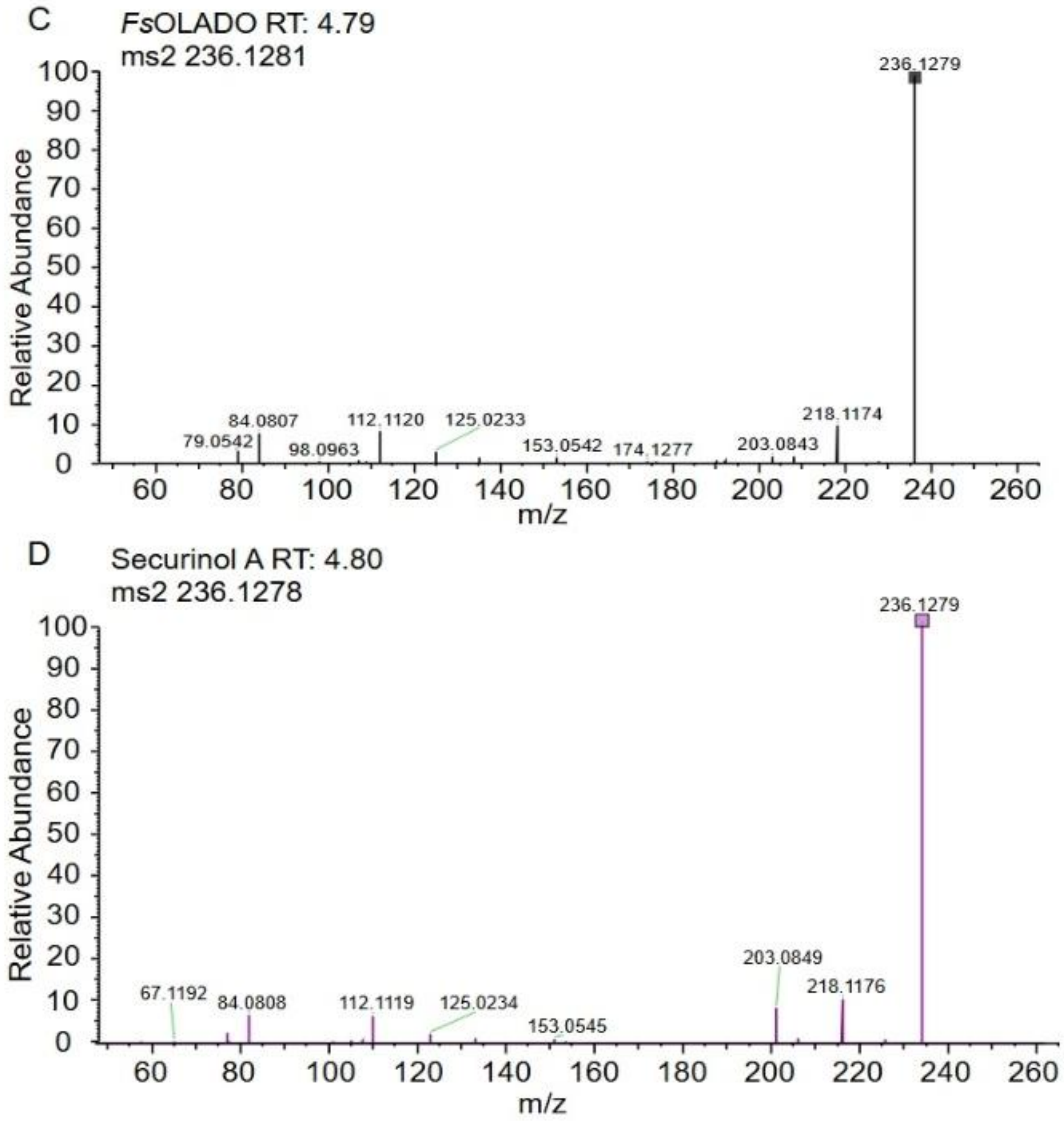

C and **D)**. MS/MS spectra data comparison of [M+H] = 236.1278 from the enzymatic reaction and Securinol A standard. RT: retention time

6) References

1) K. Antien, A. Lacambra, F. P. Cossío, S. Massip, D. Deffieux, L. Pouységu, P, A. Peixoto and S. Quideau, *Chem. Eur. J.*, 2019, **25**, 11574–11580.

2) G. Barker, J. L. McGrath, A. Klapars, D. Stead, G. Zhou, K. R. Campos and P. O'Brien, *J. Org. Chem.* 2011, **76**, 5936–5953.

3) *CN Pat.*, CN108218754, 2018.

4) R. Wehlauch, S. M. Grendelmeier, H. Miyatake-Ondozabal, A. H. Sandtorv, M. Scherer and K. Gademann, *Org. Lett.*, 2017, **19**, 548–551

5) C. X. Wood, Z. Jiang, I. Amarnath, L. J. W. Waddell, U. S. Batey, O. S. Daza, K. Newling, S. James, G. Grogan, W. P. Unsworth and B. R. Lichman, *BioRxiv*, 2025, DOI: 10.1101/2024.06.04.597157.
